# Time Toxicity in Advanced Breast Cancer: Psychological Burden, Psychiatric Vulnerability, and Implications for Patient-Centered Decision-Making

**DOI:** 10.3390/medicina62081551

**Published:** 2026-08-12

**Authors:** Giuseppe Marano, Ida Paris, Gianandrea Traversi, Osvaldo Mazza, Silvia Rotondaro, Tatiana D’Angelo, Paola Fuso, Daniela Pia Rosaria Chieffo, Gianluca Franceschini, Marianna Mazza

**Affiliations:** 1 Department of Neuroscience, Head-Neck and Chest, Section of Psychiatry, Fondazione Policlinico Universitario Agostino Gemelli IRCCS, Largo Agostino Gemelli 8, 00168 Rome, Italy; 2 Department of Neuroscience, Section of Psychiatry, Università Cattolica del Sacro Cuore, 00168 Rome, Italy; 3 Division of Gynecologic Oncology, Department of Woman and Child Health and Public Health, Fondazione Policlinico Universitario Agostino Gemelli IRCCS, 00168 Rome, Italy; 4 Unit of Medical Genetics, Department of Laboratory Medicine, Ospedale Isola Tiberina-Gemelli Isola, 00186 Rome, Italy; gianandrea.traversi@gmail.com; 5 Spine Surgery Department, Bambino Gesù Children’s Hospital IRCCS, 00168 Rome, Italy; osvaldo.mazza1973@hotmail.it; 6 Clinical Psychology Unit, Fondazione Policlinico Universitario Agostino Gemelli IRCCS, Largo Francesco Vito 1, 00168 Rome, Italy; 7 Department Women Children and Public Health, Università Cattolica del Sacro Cuore, 20123 Rome, Italy; 8 Breast Surgery Unit, Department of Woman and Child’s Health and Public Health Sciences, Fondazione Policlinico Universitario Agostino Gemelli IRCCS, 00168 Rome, Italy; 9 Department of Medical and Surgical Sciences, Catholic University of the Sacred Heart, 00168 Rome, Italy

**Keywords:** time toxicity, breast cancer, psycho-oncology, psychological distress, psychiatric vulnerability, health-related quality of life

## Abstract

The paradigm shift toward precision oncology in advanced breast cancer has increased therapeutic complexity, prolonging survival for many patients while simultaneously expanding the temporal burden associated with cancer care. Time toxicity, defined as the cumulative time spent receiving, accessing, coordinating, and recovering from medical interventions, is increasingly recognized as a clinically meaningful but still under-assessed dimension of patient-centered value. Beyond its organizational and economic implications, time toxicity may exert profound psychological and psychiatric effects, particularly in patients with advanced disease, for whom time represents not only a practical resource but also an existential, relational, and emotional domain. This narrative review examines how objective and subjective dimensions of time toxicity may inform patient-centered decision-making in advanced breast cancer. We summarize current evidence on the temporal demands of cancer care in advanced breast cancer and their psychological and relational consequences, focusing on how these factors can be discussed in patient-centered treatment decisions. From a value-based oncology perspective, the review examines how treatment optimization, subcutaneous formulations, oral therapies, telemedicine-based surveillance, decentralized care models, and early psycho-oncological assessment may reduce non-value-added time in clinical settings while preserving quality of care. Methodological challenges in measuring time toxicity and correlating it with overall survival, health-related quality of life, anxiety, depression, demoralization, and illness-related distress are also discussed. Incorporating time toxicity into shared decision-making may help clinicians move beyond traditional efficacy endpoints. It may support a more comprehensive approach in which survival benefit, psychological well-being, personal priorities, and the subjective value of time are considered together.

## 1. Introduction

Advanced breast cancer has increasingly become a paradigmatic condition in which therapeutic innovation, prolonged survival, and growing treatment complexity coexist. The development of endocrine-based combinations, targeted agents, antibody–drug conjugates, immune-based approaches in selected molecular subgroups, and increasingly individualized sequencing strategies has substantially expanded the therapeutic landscape of metastatic breast cancer [1,2,3]. However, the same progress that has improved disease control has also generated new forms of burden that are not fully captured by traditional oncological endpoints.

In clinical trials and routine oncology practice, treatment value is still predominantly assessed through metrics such as overall survival, progression-free survival, response rate, toxicity profile, and, to a lesser extent, health-related quality of life. Although these outcomes remain essential, they may insufficiently reflect the lived experience of patients with advanced disease. A regimen associated with a modest survival advantage may require frequent hospital visits, prolonged infusions, intensive monitoring, repeated imaging, management of adverse events, and substantial recovery time. Conversely, a treatment with a comparable efficacy profile may impose a lower burden on daily life if it reduces time spent in clinical settings or allows care to be delivered closer to the patient’s home [4,5].

The concept of time toxicity has emerged to describe the cumulative temporal burden generated by cancer care. Time toxicity encompasses the cumulative time patients spend receiving cancer care and managing its consequences [4]. In advanced cancer, time toxicity assumes particular relevance because time itself becomes a central dimension of care. For patients living with an incurable disease, the value of treatment cannot be reduced to the extension of chronological survival alone. The quality, meaning, and usability of time become equally important.

Despite its clinical importance, time toxicity remains under-recognized in both research and practice. Most oncology trials provide limited information on the amount of time patients spend interacting with healthcare systems, and even fewer studies assess the psychological consequences of this burden. Time toxicity has been operationalized through measures of healthcare-related time, although these indicators do not fully capture its subjective psychological meaning [6,7].

From a psychiatric and psychological perspective, time toxicity may contribute to distress, anxiety, depressive symptoms, demoralization, decisional fatigue, and loss of perceived control. Psychosocial distress is now widely recognized as a clinically relevant dimension of cancer care, and contemporary guidelines emphasize the importance of identifying and treating psychological problems across the cancer trajectory [8]. Distress may be particularly pronounced in patients with advanced breast cancer [9,10]. In addition to physical symptoms and treatment-related toxicity, patients may face uncertainty, fear of death, existential concerns, family disruption, and repeated changes in prognosis and treatment planning. The accurate identification of these symptoms is part of a broader psychiatric effort to validate specific depressive and distress syndromes across diverse clinical populations and life contexts [11].

Advanced breast cancer therefore represents a clinically relevant setting in which to reconsider how treatment value is defined. A patient-centered approach requires that survival benefit, toxicity, quality of life, psychological well-being, treatment burden, and personal priorities are evaluated together rather than separately [12]. This is particularly important when therapeutic options differ in route of administration, monitoring intensity, expected adverse events, frequency of hospital access, and impact on daily functioning.

Previous studies on time toxicity have primarily focused on measurable healthcare-related time, including treatment administration, healthcare contact days, travel, and treatment-related recovery [4,5,6,7]. However, much of this evidence derives from general oncology populations or cancer types other than advanced breast cancer. In parallel, breast cancer and psycho-oncology literature has examined psychological distress, psychiatric vulnerability, patient preferences, and psychosocial support [5,8,9,10], but these outcomes have not been consistently integrated with objective measures of time burden. This review integrates these domains into a single conceptual framework for advanced breast cancer.

The central question of this narrative review is how the objective and subjective burden of healthcare-related time can be conceptualized and incorporated into patient-centered decision-making for individuals with advanced breast cancer. To address this question, the review examines three closely related domains: the measurable time required to access, receive, and recover from cancer care; the psychological and relational meaning attributed to this time; and the clinical or organizational strategies that may reduce avoidable temporal burden. Because direct evidence on time toxicity in advanced breast cancer remains limited, this review also draws on studies from broader breast cancer, advanced cancer, and general psycho-oncology populations.

This review provides a conceptual synthesis of the available evidence to support patient-centered care and future research in advanced breast cancer.

## 2. Materials and Methods

This article was designed as a narrative review with a conceptual and clinically oriented aim: to examine how the objective and subjective burden of healthcare-related time may inform patient-centered decision-making in advanced breast cancer. Given the heterogeneity of the available evidence, a narrative review was considered the most appropriate approach. The review followed recommendations for high-quality narrative reviews [13]. Although the review was not designed as a systematic review or meta-analysis, structured search and reporting procedures were adopted, where applicable, to improve transparency and reproducibility [14].

The central review question was as follows: how can the objective and subjective burden of healthcare-related time be conceptualized and incorporated into patient-centered decision-making for individuals with advanced breast cancer? Psychological distress, psychiatric vulnerability, caregiver burden, decisional fatigue, and loss of autonomy were considered as potential modifiers or consequences of time toxicity rather than as independent primary topics.

A structured literature search was conducted in PubMed/MEDLINE, Scopus, Web of Science, and PsycINFO. The principal search period covered publications from January 2015 to 31 May 2026, reflecting the relatively recent development of time toxicity as an explicit construct in oncology. Earlier seminal publications were also considered when they introduced foundational concepts, validated clinically relevant psychological constructs, or provided theoretical models that remain relevant to contemporary psycho-oncology, palliative care, and supportive cancer care.

The search focused on publications addressing time toxicity, treatment burden, patient-reported outcomes, shared decision-making, psychological distress, psychiatric vulnerability, caregiver burden, and health-related quality of life in patients with advanced or metastatic breast cancer. When necessary, evidence from broader advanced cancer populations was included to contextualize findings.

Searches combined terms related to time toxicity, advanced breast cancer, metastatic breast cancer, treatment burden, patient-reported outcomes, shared decision-making, psycho-oncology, psychological distress, anxiety, depression, demoralization, caregiver burden, and quality of life. Reference lists of eligible articles were also screened. Reference lists of relevant reviews, guidelines, conceptual papers, and eligible primary studies were also examined to identify additional publications.

Eligible publications included original studies, qualitative studies, reviews, clinical guidelines, and conceptual papers relevant to the review objectives. Eligible studies addressed time toxicity, treatment burden, psychological outcomes, caregiver burden, patient-reported outcomes, shared decision-making, or related care models. Publications not written in English, conference abstracts, opinion papers, studies limited to early breast cancer without relevance to advanced disease, and articles lacking clinical or patient-centered relevance were excluded.

The literature selection process involved title and abstract screening followed by full-text assessment of publications considered potentially relevant to the review question. The references retained in the final manuscript represent the publications judged to be directly relevant to the conceptual synthesis.

Relevant information on study characteristics, time burden, psychological outcomes, caregiver burden, patient-reported outcomes, and clinical implications was extracted. Particular attention was given to distinguishing general psychological distress from clinically significant anxiety or depression, demoralization, existential distress, decisional fatigue, and perceived loss of autonomy. 

Because of the heterogeneity of study designs, populations, interventions, and outcome measures, no quantitative synthesis or formal meta-analysis was performed. The evidence was instead organized according to clinically relevant thematic domains: objective time burden; subjective time burden; psychological and psychiatric modifiers; caregiver and relational burden; health-related quality of life; treatment optimization; and shared decision-making. Where possible, evidence derived directly from advanced or metastatic breast cancer populations was distinguished from evidence extrapolated from broader breast cancer or general oncology settings.

Findings were synthesized into a conceptual framework distinguishing objective time burden, subjective time burden, psychological and psychiatric modifiers, caregiver burden, and the existential value of time. The framework summarizes the literature and is intended to support clinical discussion and future research.

During the preparation of this manuscript, the authors used Gemini 3.1 (Google, June 2026) for language editing, text refinement, assistance in improving clarity and readability of the manuscript, and support in the design and refinement of illustrative figures. The authors have reviewed and edited the output and take full responsibility for the content of this publication.

## 3. Defining Time Toxicity in Advanced Cancer Care

### 3.1. Conceptual Definition

Time toxicity refers to the cumulative amount of time that patients and their families spend on activities directly or indirectly related to cancer care. It includes time devoted to treatment, monitoring, healthcare contacts, travel, care coordination, adverse event management, and recovery [4,15]. It represents a burden generated not by the malignancy alone, but also by the structure, intensity, and organization of the healthcare pathway.

Patients with advanced cancer often undergo multiple treatments, repeated assessments, supportive care, and frequent healthcare contacts. Time toxicity extends beyond treatment administration and includes broader temporal demands that disrupt daily life and psychological well-being [15,16]. Most methodological studies defining healthcare contact days and related time-based outcomes have involved mixed oncology or non-breast cancer populations. Their relevance to advanced breast cancer is conceptually strong, but disease-specific validation remains limited.

In advanced cancer, treatment value should be assessed not only by survival but also by the amount and quality of time patients spend outside healthcare settings and by how they perceive this time [4,15].

### 3.2. Direct Time Costs

The most visible component of time toxicity is represented by direct time costs. Direct time costs include treatment administration, monitoring, diagnostic procedures, hospital visits, emergency care, and follow-up appointments. In clinical practice, even apparently short medical encounters may occupy an entire day when travel, waiting time, administrative procedures, pre-treatment assessments, post-treatment observation, and recovery are considered. This explains why several authors have proposed measuring time toxicity not only as treatment duration, but also as the number of days with any healthcare contact, sometimes conceptualized as “contact days” or days not spent entirely at home [6,16].

Systemic therapies often require repeated hospital attendance together with laboratory assessments, clinical evaluations, and monitoring. These visits are often accompanied by pre-treatment laboratory evaluation, clinical assessment, infusion preparation, observation for adverse reactions, and post-treatment management. Consequently, time burden depends not only on treatment schedules but also on toxicity, monitoring requirements, and unplanned healthcare use [17].

Treatments with similar efficacy may impose substantially different temporal burdens, which should be considered during shared decision-making [18].

### 3.3. Indirect Time Costs

Indirect time costs include travel, transportation, work absence, childcare, administrative activities, caregiver coordination, and post-treatment recovery. For many patients, particularly those living far from oncology centers, receiving treatment involves not only the scheduled appointment but also a complex chain of logistical activities before and after the clinical encounter.

Indirect time costs may be particularly burdensome for patients with limited socioeconomic resources, rural residence, reduced mobility, low social support, caregiving responsibilities, or financial insecurity. In such cases, time toxicity intersects with financial toxicity and social vulnerability. Time spent travelling, waiting, or coordinating care can translate into lost income, dependence on family members, caregiver strain, and reduced access to meaningful activities. This burden is not evenly distributed across patient populations; rather, it may disproportionately affect individuals who already experience structural barriers to care [19].

Caregivers also experience substantial time burden through transportation, symptom monitoring, medication management, and coordination of care, with important consequences for family functioning and caregiver well-being [20].

### 3.4. Hidden and Subjective Time Costs

Beyond measurable healthcare utilization, time toxicity also includes subjective psychological burden. This dimension includes anticipatory anxiety, uncertainty, fear of progression, rumination, and treatment-related hypervigilance.

The phenomenon commonly referred to as “scanxiety” illustrates this subjective component. Imaging assessments may take only a limited amount of chronological time, but their psychological impact can extend over days or weeks before and after the examination. Patients may experience anxiety while waiting for scans, distress while anticipating results, and emotional destabilization after receiving ambiguous or unfavorable findings. Recent research confirms that scan-related anxiety and fear of cancer recurrence or progression are clinically relevant constructs in cancer populations and may influence quality of life, emotional functioning, and healthcare utilization [21,22]. In advanced disease, this anxiety is not simply fear of recurrence, but often fear of progression, treatment failure, loss of future options, and approaching mortality.

Subjective time toxicity may also involve a loss of spontaneity and perceived autonomy. Even when patients are not physically in hospital, they may feel unable to plan travel, family events, work commitments, or personal projects because their lives are structured around treatment cycles, scan intervals, laboratory checks, and the possibility of sudden clinical deterioration. This creates a form of psychological occupation of time: the disease and its treatment colonize not only the calendar, but also imagination, attention, and future-oriented thinking.

### 3.5. Time Toxicity as a Patient-Centered Outcome

Defining time toxicity as a patient-centered outcome has important implications for advanced cancer care. Time toxicity complements traditional oncological outcomes by capturing how patients experience healthcare-related time.

It is particularly relevant when treatments offer similar efficacy but differ in administration, monitoring, or recovery burden [5,23].

In advanced breast cancer, time toxicity should be interpreted in light of psychological vulnerability, social support, personal values, and treatment goals. Because it encompasses measurable healthcare utilization and subjective disruption of daily life, its multidimensional nature is summarized in Table 1.

## 4. The Psychological Meaning of Time in Advanced Breast Cancer

### 4.1. From Chronological Time to Lived Time

In advanced breast cancer, time is not only a chronological variable but also a psychological, relational, and existential dimension. Oncology traditionally measures time through objective indicators such as overall survival, progression-free survival, time to progression, duration of response, treatment intervals, and follow-up schedules. These measures are indispensable for evaluating treatment efficacy, but they do not fully capture how patients experience time while living with an incurable disease. From a psychological perspective, the same amount of chronological time may be perceived as meaningful, empty, suspended, threatening, or fragmented, depending on the patient’s emotional state, prognosis awareness, symptom burden, social context, and perceived control.

This distinction between chronological time and lived time is particularly relevant in advanced cancer. Previous research has shown that patients with advanced cancer may experience altered time perception, including the perception that time moves more slowly, and that such changes are associated with distress [24]. More recent work has further emphasized that cancer treatment may reorganize patients’ temporal experience around treatment cycles, appointments, scan intervals, and periods of waiting. Chemotherapy and systemic therapy schedules may become a kind of “treatment clock”, structuring the patient’s week, month, and future expectations around the rhythm of care rather than around personal priorities [25]. These observations derive largely from advanced cancer and mixed oncology populations rather than exclusively from patients with advanced breast cancer. They are therefore used here to inform the conceptual interpretation of lived time, rather than as evidence of a breast cancer-specific phenomenon.

### 4.2. Time, Autonomy, and Loss of Control

A central psychological consequence of time toxicity is the loss of perceived control. Advanced breast cancer may impose a clinical rhythm that competes with the patient’s own rhythm of life. Treatment appointments, infusion schedules, laboratory monitoring, radiological evaluations, toxicity management, and unexpected clinical events may progressively reduce the patient’s ability to plan work, family activities, travel, intimacy, rest, and meaningful personal projects. Even when the patient is not physically in a healthcare facility, the anticipation of future appointments or possible deterioration may limit the subjective freedom to inhabit the present.

This loss of control is clinically relevant because perceived control is closely linked to psychological adjustment in serious illness. Patients may experience a shift from being active agents of their lives to becoming subjects organized around medical demands. In advanced cancer, this may generate frustration, helplessness, anger, sadness, or resignation. Time becomes something allocated by the healthcare system rather than something lived according to personal values.

Many patients continue to occupy multiple social and relational roles, including partner, mother, worker, caregiver, daughter, friend, and community member. When cancer care repeatedly interrupts these roles, the psychological burden may extend beyond inconvenience [26]. It may affect identity, dignity, and the continuity of self.

### 4.3. The Future Horizon and Existential Uncertainty

Advanced breast cancer profoundly modifies the patient’s relationship with the future. For many patients, the future becomes both precious and uncertain. Treatment may offer disease control, symptom relief, or survival prolongation, but it may also reinforce awareness of incurability, mortality, and dependence on ongoing medical intervention.

Patients with advanced cancer frequently face concerns related to death, loss of meaning, dignity, dependency, unfinished life projects, family separation, and fear of suffering. Existential distress has been increasingly recognized as a clinically significant dimension of advanced cancer care, encompassing demoralization, death anxiety, loss of purpose, hopelessness, and dignity-related distress [27,28]. In this context, time toxicity may intensify existential distress when patients perceive that a substantial portion of their remaining time is spent in hospitals, waiting rooms, treatment units, or recovery from adverse events rather than in personally meaningful activities.

The temporal structure of advanced breast cancer may also create a condition of suspended future. Patients may hesitate to make plans because future availability is uncertain. Travel, family events, work decisions, relational commitments, and personal goals may be postponed until the next scan, the next consultation, or the next treatment response assessment. This repeated postponement may generate a psychological state in which life is experienced as provisional.

### 4.4. Identity, Meaning, and the Reorganization of Priorities

The diagnosis and treatment of advanced breast cancer may lead patients to reconsider their identity, priorities, and sense of meaning. For some patients, the awareness of limited time may generate a process of reprioritization, strengthening relationships, increasing focus on meaningful activities, and clarifying personal values. For others, the same awareness may produce loss of meaning, demoralization, withdrawal, or a sense that the future has been prematurely closed.

Studies on long-term living with advanced cancer describe a complex combination of adaptation and burden [29]. Patients may experience uncertainty, altered identity, social disruption, fear of progression, and dependence on treatment. They must also integrate ongoing systemic therapy into daily life. In breast cancer specifically, existential concerns may be closely linked to body image, femininity, sexuality, fertility, motherhood, relational roles, and continuity of personal identity. Research on breast cancer patients with fertility-related concerns, although not limited to advanced disease, has shown how cancer can threaten meaning, identity, and future-oriented life narratives, while also leading some women to re-evaluate priorities and reconstruct meaning [30].

Time toxicity intersects with these processes because it affects the space available for identity-preserving activities.

### 4.5. Waiting, Scanxiety, and the Emotional Occupation of Time

Waiting is one of the most psychologically charged forms of time toxicity. Patients with advanced breast cancer may spend substantial time waiting for appointments, treatment administration, laboratory results, imaging procedures, scan interpretation, treatment decisions, and confirmation of disease response or progression. Although waiting time may appear administratively minor, psychologically it may be experienced as a period of heightened vulnerability.

Scan-related anxiety is a clear example of how medical time extends beyond the procedure itself. The psychological burden of imaging may begin days or weeks before the scan and continue until results are communicated and interpreted. During this interval, patients may experience intrusive thoughts, sleep disturbance, irritability, somatic hypervigilance, catastrophic expectations, and fear of progression. In advanced disease, this anxiety is not simply fear that cancer may return, but fear that the disease is progressing, that treatment is failing, or that therapeutic options are narrowing. Recent studies have highlighted the clinical relevance of scanxiety, fear of recurrence, and fear of progression across cancer populations, including breast cancer [21,22]. However, the available evidence is heterogeneous with respect to cancer type and disease stage. Its application to advanced breast cancer should therefore be interpreted as partly disease-specific and partly extrapolated from broader psycho-oncology research.

**Figure 1:**
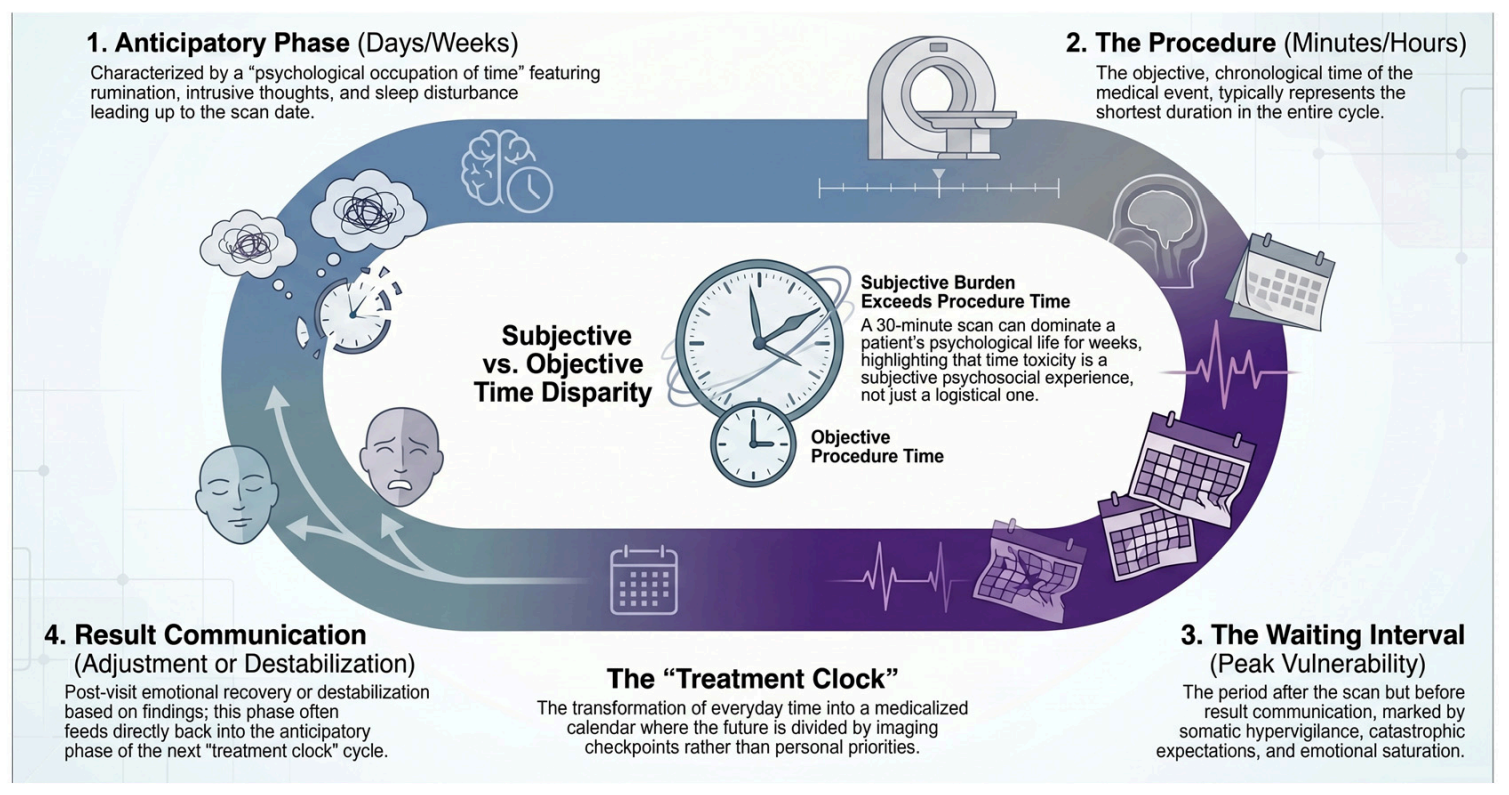
The emotional and temporal cycle of scan-related anxiety. Note: The figure schematically depicts common phases reported in the literature and in clinical practice (anticipation of imaging, waiting for the examination, waiting for results, and emotional reaction after results). It is intended as a visual summary of the text, not as a diagnostic algorithm.

Waiting also creates a subjective expansion of medical time. A scan that lasts thirty minutes may dominate the patient’s psychological life for much longer. Similarly, a consultation may occupy not only the time spent in the clinic, but also the days of anticipation before it and the emotional recovery afterward. Figure 1 schematically summarizes the typical phases of scan-related anxiety described in this section, from the scheduling of imaging to the reception of results.

### 4.6. The Value of Time as a Clinical Preference

The psychological meaning of time is not uniform across patients. Some patients may willingly accept intensive treatment schedules if they perceive them as offering hope, control, or meaningful survival gain. Others may prioritize time at home, relational presence, reduced hospital attendance, or preservation of daily routines, even when this implies accepting a less intensive treatment strategy. These differences reflect variations in values, coping style, illness understanding, risk tolerance, and existential priorities. For this reason, time should be explicitly explored as a patient preference in advanced breast cancer decision-making. Clinicians should not assume that all patients value survival extension and treatment convenience in the same way. The subjective value of time may change across the disease trajectory: a patient may initially prioritize maximal disease control, later prioritize symptom relief and autonomy, and eventually prioritize time at home or relational closure. These shifts are part of the psychological adaptation to advanced illness [31,32].

A psychiatry- and psychology-informed approach can help clinicians identify how emotional distress, depression, anxiety, demoralization, cognitive overload, family pressure, or unrealistic expectations may influence the patient’s evaluation of time. At the same time, it can protect against the opposite risk: dismissing a patient’s preference for less burdensome care as avoidance or resignation.

## 5. Psychiatric Vulnerability and Time Toxicity

### 5.1. Psychiatric Vulnerability as a Moderator of Time Toxicity

Psychiatric vulnerability may act as a moderator between objective time burden and subjective suffering [15]. This perspective is consistent with a broader clinical recognition that psychiatric symptoms may affect the experience and management of systemic illness, including communication, adherence, symptom reporting, functional status, and healthcare utilization [33]. It does not imply that psychiatric disorders directly alter the biological course of breast cancer.

Psychological distress is highly prevalent among women with breast cancer and appears to be particularly relevant in advanced disease. A recent systematic review and meta-analysis reported a pooled prevalence of psychological distress of approximately 52% among adult female breast cancer patients, with late-stage tumor and history of depression among the independent risk factors [34]. These findings are clinically important because they suggest that patients with advanced disease and prior psychiatric vulnerability may enter complex oncology pathways already at increased risk of emotional dysregulation, reduced coping capacity, and impaired adaptation.

**Figure 2:**
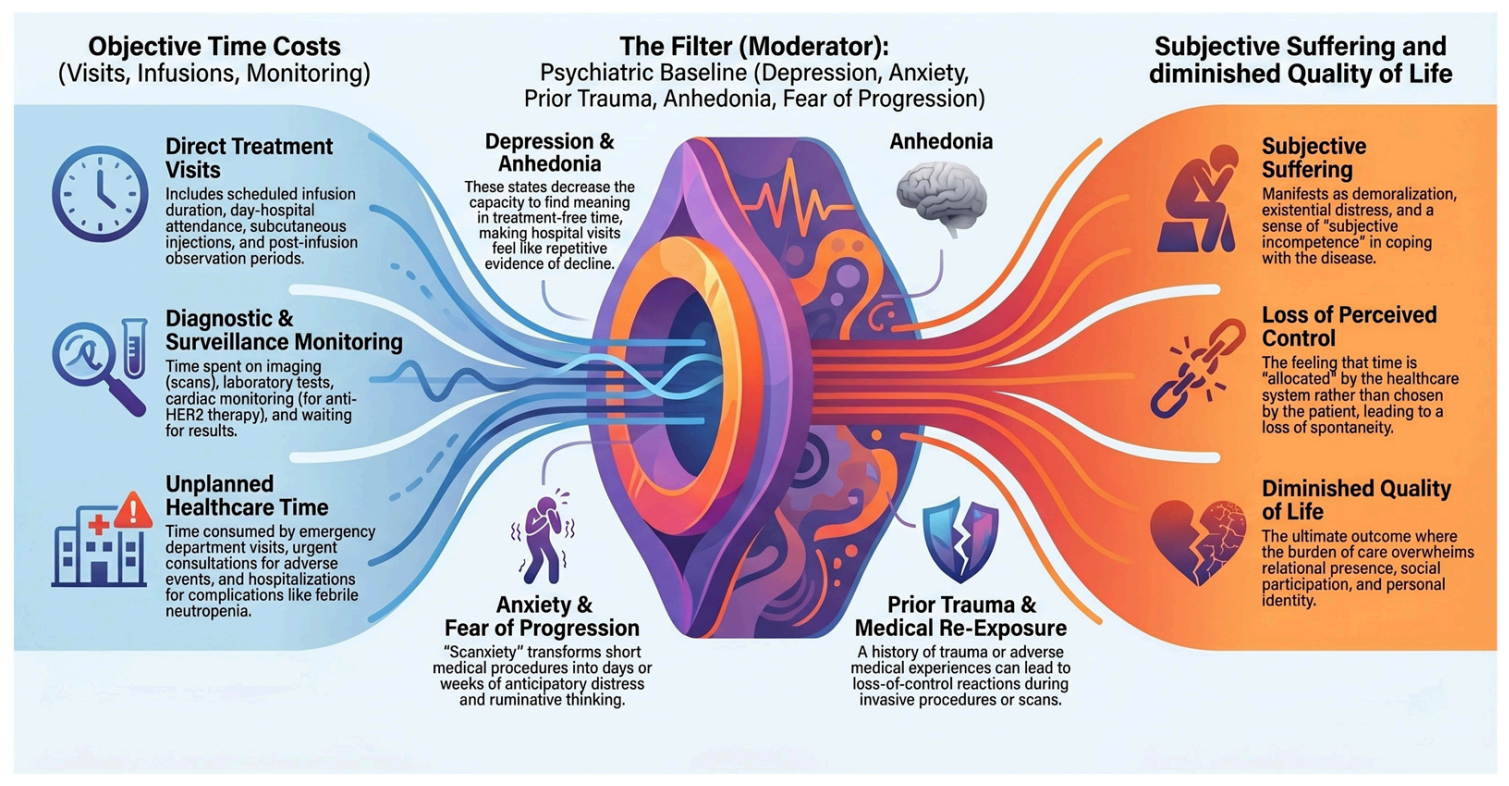
Psychiatric vulnerability and its interaction with time toxicity. Note: The figure summarizes relationships discussed in the text between baseline psychiatric conditions, distress during treatment, and perceived time burden. It is meant as a clinical schema to support discussion and hypothesis generation, not as a formal assessment tool or scoring system.

From such perspective, time toxicity may be conceptualized as a stressor whose impact depends on the patient’s psychological baseline. For a patient with good coping resources and strong social support, frequent clinical contacts may be experienced as burdensome but manageable. For a patient with depression, anxiety, demoralization, trauma history, cognitive overload, or poor support, the same temporal demands may become destabilizing. The complex interplay between objective healthcare utilization and the patient’s lived experience is further elucidated in Figure 2, which provides a schematic overview of how psychiatric vulnerability may interact with objective and subjective components of time burden in advanced breast cancer.

### 5.2. Depression, Anhedonia, and the Perception of Treatment Burden

Depression is one of the most clinically relevant forms of psychiatric vulnerability in cancer care. In breast cancer, observational evidence has linked depression and anxiety with less favorable clinical outcomes. A systematic review and meta-analysis including more than 280,000 patients reported associations between depression and recurrence, all-cause mortality, and cancer-specific mortality, as well as between anxiety and recurrence and all-cause mortality [35]. These findings should not be interpreted as evidence that psychiatric symptoms directly influence tumor biology, treatment sensitivity, recurrence, or survival. The reported associations may be partly explained by disease burden, functional impairment, frailty, comorbidity, socioeconomic disadvantage, access to care, treatment adherence, and other measured or unmeasured confounding factors. Depression and anxiety remain clinically relevant because they may affect quality of life, symptom perception, communication, treatment engagement, decision-making, adherence, and healthcare utilization [36]. Research on the gut–brain axis and microbiota may contribute to understanding mood symptoms and stress-related processes [37,38], but it does not currently establish a direct pathway from psychiatric disorders to breast cancer progression or oncological outcomes.

Depression may increase the subjective burden of time toxicity through several psychological and behavioral pathways. Anhedonia and reduced motivation may decrease the patient’s capacity to experience treatment-free time as restorative or meaningful. Psychomotor slowing, fatigue, sleep disturbance, and cognitive symptoms may make travel, waiting, treatment administration, and recovery periods more exhausting. Depressive hopelessness may alter the perceived value of treatment-related time: the patient may experience hospital visits not as an investment in survival or disease control, but as repetitive evidence of decline, dependence, and loss of future.

Depression may also be associated with difficulties in treatment adherence, communication, and shared decision-making. Some patients with clinically significant depressive symptoms may find it more difficult to process complex medical information, ask questions, compare treatment options, or express preferences. These effects are variable and should not be assumed in every patient. In the context of advanced breast cancer, unrecognized depression may therefore reduce the patient’s ability to participate fully in repeated treatment decisions [39].

The complaint may reflect a depressive syndrome, demoralization, fatigue, uncontrolled symptoms, or a mismatch between treatment demands and personal values. A psychiatric evaluation can help distinguish between these possibilities and guide appropriate intervention.

### 5.3. Anxiety, Fear of Progression, and Anticipatory Time

Anxiety is another major psychiatric dimension that may intensify time toxicity. In advanced breast cancer, anxiety is frequently linked to uncertainty, treatment response, adverse events, disease monitoring, bodily sensations, and fear of progression. Fear of progression has been described as one of the most common and burdensome psychological problems among breast cancer patients, with negative effects on quality of life and functioning [40,41]. In advanced disease, fear of progression is not a hypothetical concern about future recurrence, but a concrete fear that current treatment may stop working, that options may become limited, or that functional decline may accelerate.

Anxiety transforms time by making it anticipatory. The patient may suffer not only during the hospital visit or scan itself, but also in the days or weeks before the event. Treatment cycles, laboratory controls, and radiological reassessments can create recurrent peaks of anxiety. The period between the scan and the communication of results may become emotionally saturated, with intrusive thoughts, insomnia, irritability, somatic hypervigilance, and catastrophic expectations [42].

Higher levels of anxiety and fear of progression have also been associated with patterns of increased healthcare use in some studies. Patients may seek additional reassurance, request more frequent contact with clinicians, or experience difficulty tolerating surveillance intervals. These behaviors are influenced by multiple clinical and contextual factors and should not be attributed to anxiety alone. Patients with high fear of progression may seek additional reassurance, request more frequent contact with clinicians, monitor bodily symptoms excessively, or experience difficulty tolerating surveillance intervals. In some cases, healthcare contact may temporarily reduce anxiety; in others, it may reinforce hypervigilance and dependence on medical reassurance. Time toxicity and anxiety may therefore form a reciprocal loop: care-related time increases anxiety, and anxiety increases the perceived need for additional care-related time.

A patient with high anxiety may prefer intensive monitoring because it feels safer, even if it increases time toxicity. Another patient may experience frequent monitoring as intrusive and destabilizing. Neither response is inherently irrational. Both require exploration of the emotional meaning attributed to medical contact, uncertainty, and control.

### 5.4. Demoralization, Hopelessness, and Existential Burden

Unlike major depression, demoralization is characterized primarily by helplessness, loss of meaning, subjective incompetence, disheartenment, and difficulty coping with a stressful situation. Contemporary systematic reviews emphasize that demoralization is a clinically relevant psychological state across medical and psychiatric settings and that validated instruments, such as the Demoralization Scale and Diagnostic Criteria for Psychosomatic Research, can support its assessment [43]. In advanced cancer, demoralization may overlap with, but remain distinguishable from, depression. The evidence on demoralization derives primarily from advanced cancer and palliative care populations rather than specifically from advanced breast cancer. Its inclusion in the present model therefore represents a clinically plausible extrapolation that requires breast cancer-specific investigation.

Time toxicity may contribute to demoralization when patients perceive that treatment is no longer compatible with a meaningful life. Repeated hospital visits, cumulative adverse events, uncertain benefits, and progressive narrowing of personal activities may generate a sense that the patient is losing not only health, but also agency, dignity, and purpose. In such circumstances, the patient may ask whether the time invested in treatment is proportionate to the time and quality of life gained [44].

Demoralization may also alter the interpretation of medical time. For a demoralized patient, waiting rooms, infusion chairs, and recurrent scans may become symbols of dependency and existential defeat. The patient may not necessarily wish to die, but may feel unable to continue living under the current conditions of care. This distinction is clinically important because demoralization may be associated with desire for hastened death, suicidal ideation, and requests for treatment withdrawal, while requiring different therapeutic responses from those used for major depressive disorder [45].

The appropriate response is not simply to encourage persistence or reduce appointment frequency, but to explore meaning, goals, dignity, perceived burden, family concerns, and existential suffering. Interventions such as meaning-centered psychotherapy, dignity therapy, supportive-expressive approaches, and early palliative care may be particularly relevant when time toxicity has become existentially burdensome.

### 5.5. Decisional Fatigue and Cognitive Overload

Advanced breast cancer often requires repeated and complex decisions. Patients may need to choose between treatment lines, routes of administration, expected toxicities, monitoring requirements, trial participation, dose reductions, supportive care options, and transitions toward palliative or comfort-focused care. Each decision may involve uncertainty, probabilistic information, emotional pressure, family expectations, and fear of making the wrong choice. Over time, this process may generate decisional fatigue.

Decision fatigue is increasingly recognized in oncology, particularly among patients facing recurrence or non-curative treatment contexts. A recent qualitative study of patients with recurrent cancer found that decision fatigue affected both treatment-related decisions and daily life choices, and that patients expected clinicians to reduce this burden through clear information, empathy, and sustained communication [46]. Because this evidence was obtained in recurrent cancer populations rather than exclusively in advanced breast cancer, its relevance to the present review is interpretive. Nevertheless, repeated treatment sequencing and recurrent preference-sensitive decisions make decision fatigue a clinically plausible concern in advanced breast cancer.

Time toxicity contributes to decisional fatigue because treatment burden is not limited to physical attendance. Patients must continually organize time, compare options, anticipate side effects, plan around appointments, coordinate family responsibilities, and decide how much of their remaining time should be devoted to treatment.

Cognitive overload may be further exacerbated by depression, anxiety, fatigue, sleep disruption, pain, and treatment-related cognitive symptoms. A psychologically informed shared decision-making process should therefore reduce unnecessary cognitive burden by presenting information clearly, eliciting values explicitly, involving caregivers when appropriate, and allowing adequate time for reflection without abandoning the patient to unsupported choice.

### 5.6. Trauma, Prior Psychiatric History, and Medical Re-Exposure

A history of psychiatric illness, trauma, or previous adverse medical experiences may shape how patients experience cancer-related time. For some patients, repeated exposure to hospital environments, invasive procedures, scans, needles, waiting rooms, alarms, clinical language, and bodily monitoring may reactivate prior vulnerability. In such cases, time toxicity is not only cumulative but also associative: the healthcare setting may become linked with fear, loss of control, bodily threat, or past traumatic memories. Evidence linking prior trauma or psychiatric history with the experience of repeated cancer care is drawn mainly from general psycho-oncology and medical trauma literature. Direct studies examining these relationships in advanced breast cancer remain limited.

Patients with prior depression, anxiety disorders, post-traumatic symptoms, eating disorders, substance use disorders, or severe mental illness may require specific attention. Standard oncology pathways are often designed around disease stage and treatment protocol, but not necessarily around psychiatric vulnerability. Yet a patient with panic disorder may experience infusion units or radiology suites differently from a patient without anxiety. A patient with trauma history may experience loss of bodily control during procedures as particularly distressing. A patient with severe depression may find the logistics of care disproportionately exhausting [47].

This perspective argues for more individualized support so that treatment can remain feasible, tolerable, and aligned with the patient’s values. Psychiatric assessment may identify modifiable factors such as untreated depression, panic symptoms, insomnia, medication interactions, substance use, cognitive impairment, or inadequate social support. Addressing these factors may reduce the subjective burden of time toxicity and improve the patient’s ability to engage in care [48].

### 5.7. Clinical Implications: Screening and Stratification

The relationship between psychiatric vulnerability and time toxicity has practical implications. Distress screening should not be limited to the initial diagnosis or disease progression. It should be repeated at clinically meaningful transition points, including treatment initiation, therapy change, progression, recurrence, hospitalization, toxicity-related dose modification, and discussions about goals of care. Contemporary guidelines already emphasize the need to recognize distress as a clinically relevant dimension across the cancer trajectory [8].

Patients at high psychiatric risk should be identified before treatment burden becomes intolerable. Relevant risk factors include prior psychiatric history, current depression or anxiety symptoms, high fear of progression, poor social support, caregiver strain, financial insecurity, high geographic distance from care, cognitive difficulties, insomnia, pain, fatigue, and limited understanding of prognosis. Most of these risk factors are supported by broader psycho-oncology evidence and should not yet be considered a validated risk-stratification system for time toxicity in advanced breast cancer.

Treatment discussions should include explicit questions about time, control, and emotional sustainability [49]. Clinicians may ask how much time the patient is willing to spend in hospital and which activities she most wants to preserve. They may also explore whether clinical visits feel reassuring or intrusive, how the patient experiences waiting for scans, and whether treatment schedules are compatible with her psychological and relational needs.

Psychiatric vulnerability may influence how objective time burden is perceived, tolerated, and integrated into treatment decisions.

Importantly, the relationships discussed in this section should be interpreted primarily as clinical and observational associations. Current evidence does not demonstrate that depression, anxiety, demoralization, trauma-related symptoms, or other psychiatric conditions directly modify tumor biology, treatment sensitivity, disease progression, recurrence, or survival in advanced breast cancer. Their principal established relevance concerns psychological well-being, quality of life, symptom perception, communication, treatment engagement, adherence, decision-making, functional status, caregiver relationships, and healthcare utilization. Observed associations with oncological outcomes may reflect complex interactions among disease severity, frailty, comorbidity, socioeconomic conditions, access to care, treatment exposure, and other confounding variables.

The main psychiatric and psychological factors that may amplify the subjective burden of time toxicity are summarized in Table 2.

## 6. Measuring Time Toxicity: Clinical and Methodological Challenges

### 6.1. The Need for Standardized Measurement

Although time toxicity is increasingly recognized as a clinically meaningful dimension of cancer care, its measurement remains methodologically challenging. Unlike traditional oncological endpoints, such as overall survival, progression-free survival, response rate, or adverse event grading, time toxicity does not yet have a universally accepted definition, standardized metric, or validated measurement framework.

A recent scoping review of prospective oncological studies showed that time toxicity is still infrequently reported in cancer clinical trials and that available studies use heterogeneous definitions and metrics [50]. Few available measures have been developed or validated specifically for advanced breast cancer. Consequently, the proposed combination of healthcare utilization metrics and patient-reported assessments should be viewed as a research-oriented approach rather than an established disease-specific measurement standard.

A valid measurement strategy should distinguish between planned and unplanned care, inpatient and outpatient contact, in-person and remote encounters, treatment-related and disease-related time, and objective versus subjective time burden. Without such distinctions, time toxicity risks becoming an imprecise label rather than a clinically useful endpoint.

### 6.2. Contact Days and Home Days

Among available metrics, the concept of healthcare contact days has gained particular attention. A contact day is generally defined as any day on which the patient has in-person contact with the healthcare system, including outpatient visits, treatment administration, laboratory tests, imaging, emergency department visits, and hospital admissions. This approach has the advantage of being patient-centered and pragmatic, because even a brief healthcare encounter may disrupt the whole day when travel, waiting, preparation, and recovery are considered [6].

Contact days can be complemented by the concept of home days, defined as days without in-person healthcare contact. Home days may be especially relevant in advanced cancer because they represent time potentially available for family life, rest, personal activities, social roles, and psychological recovery. In this respect, home days are not merely the inverse of contact days, but a clinically meaningful indicator of time preserved outside the healthcare system.

It should be noted that contact days and home days have important limitations. They are usually based on administrative data or trial records and may not capture travel time, waiting time, caregiver time, symptom management at home, telephone triage, pharmacy visits, or emotional recovery. They usually treat each contact day equally, although the burden of a hospitalization day, a chemotherapy infusion day, a 15-min teleconsultation, and a routine blood test may differ substantially. They may not distinguish between goal-concordant and non-goal-concordant healthcare contact. Some patients may value frequent visits because they provide reassurance, perceived safety, or emotional support, whereas others may experience the same visits as intrusive and exhausting.

Recent work in advanced non-small cell lung cancer has shown that contact days can be extracted from trial and routine-care data and may provide clinically relevant information about treatment burden [51]. Nevertheless, these studies also highlight that contact days should be interpreted in relation to survival duration, treatment phase, disease trajectory, and healthcare setting. Expressing contact days as an absolute number may be misleading if patients differ substantially in survival time. For this reason, some authors have expressed time toxicity as a proportion of days alive spent in contact with the healthcare system.

### 6.3. Planned Versus Unplanned Time Toxicity

A further methodological challenge is the distinction between planned and unplanned time toxicity. Planned time toxicity includes scheduled visits, infusions, radiological assessments, laboratory monitoring, and protocol-mandated procedures. Unplanned time toxicity includes urgent consultations, emergency department visits, hospital admissions, unscheduled imaging, additional laboratory tests, toxicity management, and complications requiring clinical reassessment.

Planned time toxicity may reflect the expected burden of a treatment regimen or trial protocol. It may be anticipated, discussed, and incorporated into shared decision-making. Unplanned time toxicity, by contrast, often reflects adverse events, disease progression, comorbidity, inadequate symptom control, or gaps in supportive care. It may be more distressing because it disrupts the patient’s sense of predictability and control [52].

In advanced breast cancer, a treatment schedule may appear acceptable when only planned visits are considered, but become much more burdensome when neutropenia, diarrhea, fatigue, pain, infections, infusion reactions, emergency assessments, or hospitalizations are included. Therefore, measuring time toxicity only through protocol-defined visits may underestimate the real-world burden of care. Conversely, measuring only hospitalizations may underestimate the cumulative burden of ambulatory treatment, monitoring, and repeated short encounters.

Future studies should therefore report planned and unplanned time toxicity separately. This would allow clinicians to explain not only how often a patient is expected to attend the hospital, but also how frequently additional healthcare contact may occur because of toxicity or complications. Such information would be particularly useful when comparing treatments with similar efficacy but different safety profiles, monitoring requirements, and routes of administration.

### 6.4. The Problem of Intensity and Quality of Time

Another major limitation of current measurement approaches is that they often quantify the presence or absence of healthcare contact without assessing the intensity or quality of that time. A contact day may include a short blood test, a prolonged infusion, a complex multidisciplinary visit, a radiological examination, an emergency department stay, or an inpatient admission. These events are not equivalent from the patient’s perspective.

The intensity of time toxicity depends on several factors: duration of the encounter, physical discomfort, waiting time, travel distance, uncertainty, emotional salience, need for caregiver accompaniment, financial consequences, and recovery afterward [53].

For example, a scan appointment may be brief but emotionally intense because of fear of progression. A telemedicine visit may reduce travel time but still occupy psychological space if it concerns disease progression or treatment failure. Similarly, an infusion day may be experienced as tolerable if the patient feels supported, or as exhausting if it is accompanied by anxiety, fatigue, nausea, or prolonged waiting.

This problem is central to a psychiatry- and psychology-informed approach. Two patients with the same number of contact days may report very different levels of burden. This variability may depend on depression, anxiety, demoralization, coping style, social support, illness understanding, and personal values. As a result, measurement frameworks should combine objective indicators with patient-reported outcomes.

### 6.5. Patient-Reported Outcomes and Subjective Time Burden

Patient-reported outcomes are essential for measuring the subjective dimension of time toxicity. Existing instruments such as the EORTC QLQ-C30, disease-specific breast cancer modules, distress screening tools, fear of progression scales, and patient-reported adverse event systems can capture important aspects of quality of life, symptoms, emotional functioning, and treatment burden [54].

The Patient-Reported Outcomes version of the Common Terminology Criteria for Adverse Events, known as PRO-CTCAE, provides a validated method for capturing symptomatic adverse events directly from patients [55]. PRO-CTCAE is highly relevant to time toxicity because symptoms such as fatigue, nausea, insomnia, anxiety, neuropathy, pain, and cognitive difficulties may determine recovery time after treatment and influence the perceived burden of healthcare contact. PRO-CTCAE does not directly measure time lost to care, waiting, travel, or the psychological occupation of time.

Therefore, time toxicity measurement should include specific patient-reported items addressing temporal burden. Assessment may first address the time spent travelling, waiting, and recovering after treatment. Additional questions may explore whether appointments interfere with meaningful activities or limit future planning. Clinicians should also examine whether medical care generates anxiety before or after visits and whether the patient considers the time spent in care worthwhile. Such measures would allow researchers to distinguish between objective time exposure and subjective time burden.

A psychiatry-informed assessment should also include emotional correlates of time toxicity, including anxiety, depression, demoralization, fear of progression, decisional fatigue, perceived loss of autonomy, and caregiver strain. Without these measures, time toxicity may be reduced to an administrative metric and fail to capture its psychological impact.

### 6.6. Capturing Telemedicine and Digital Care

The expansion of telemedicine has further complicated the measurement of time toxicity. Telemedicine may reduce travel time, waiting-room exposure, caregiver burden, and disruption of daily activities. However, it does not eliminate time toxicity. Remote visits still require scheduling, preparation, emotional engagement, cognitive effort, and sometimes additional coordination with local laboratories, pharmacies, or imaging centers.

Recent evidence suggests that modern time toxicity measures should include both in-person and telehealth interactions [56]. This is particularly important because a reduction in in-person visits may not necessarily translate into a reduction in overall healthcare-related time. Telehealth may replace some visits, add new contacts, shift responsibilities to patients and caregivers, or increase the amount of home-based medical work. For example, patients may need to measure symptoms, manage digital platforms, coordinate local tests, upload documents, or communicate asynchronously with clinical teams.

Telemedicine should not be automatically interpreted as a reduction in time toxicity. Its impact depends on whether it replaces unnecessary visits, improves access, reduces travel, and preserves patient autonomy, or whether it adds another layer of care coordination [57]. Measurement frameworks should therefore distinguish between in-person contact days, telehealth contact days, asynchronous digital communication, home monitoring, and patient/caregiver administrative workload.

### 6.7. Data Sources and Methodological Trade-Offs

Different data sources capture different aspects of time toxicity. Clinical trial protocols can provide detailed information on scheduled visits, treatment administration, imaging frequency, laboratory monitoring, and adverse event assessments. However, they may not capture real-world delays, travel burden, caregiver time, or unscheduled care outside the trial site. Administrative data can capture hospitalizations, emergency visits, outpatient contacts, and claims-based healthcare utilization, but may lack information on waiting time, emotional burden, symptom recovery, and patient priorities [58].

Electronic health records can provide granular information on appointments, orders, medications, procedures, and hospital encounters, but their completeness depends on documentation quality and system integration. Patient diaries and ecological momentary assessment may capture subjective experience and daily disruption more accurately, but they increase respondent burden and may be difficult to implement in advanced cancer populations. Wearable devices and digital tools may help measure activity, fatigue, sleep, and recovery patterns, but they do not replace subjective reports of meaning, distress, and perceived loss of time.

A robust measurement strategy should therefore be multimodal. It should combine administrative or electronic healthcare data with patient-reported outcomes, caregiver-reported burden, and contextual information such as distance from care, transportation needs, socioeconomic status, and social support.

### 6.8. Interpretation and Clinical Meaningfulness

Finally, a major challenge is determining what level of time toxicity is clinically meaningful. Unlike survival endpoints, where differences in months or hazard ratios can be interpreted within established trial frameworks, time toxicity lacks widely accepted thresholds. It remains unclear how many contact days constitute excessive burden, how much reduction is meaningful to patients, or how time toxicity should be weighed against survival benefit, symptom control, and treatment-related toxicity.

The clinical meaning of time toxicity is also preference-sensitive. Some patients may accept high time burden for a small probability of benefit, while others may prioritize time at home even when additional therapy is available. Therefore, time toxicity cannot be interpreted without reference to patient values. A treatment associated with many contact days may be acceptable if it provides meaningful symptom relief, substantial disease control, or reassurance. Conversely, a treatment with fewer contact days may not be preferable if it increases anxiety, shifts excessive responsibility to the patient, or compromises perceived safety.

In advanced breast cancer, the most useful approach may be to present time toxicity alongside traditional outcomes. For example, treatment discussions could include expected survival benefit, probability of response, adverse events, visit frequency, estimated contact days, likelihood of hospitalization, recovery time, and patient-reported burden [23]. Such multidimensional information would allow clinicians and patients to evaluate not only whether a treatment is effective, but whether its temporal, emotional, and relational costs are proportionate to the expected benefit.

## 7. Time Toxicity and Treatment Burden in Advanced Breast Cancer

### 7.1. Treatment Burden as a Multidimensional Construct

Advanced breast cancer is no longer defined by a single therapeutic pathway. Contemporary management is characterized by biomarker-driven sequencing, repeated reassessment of tumor biology, prolonged systemic therapy, and individualized combinations of endocrine therapy, targeted agents, chemotherapy, antibody–drug conjugates, anti-HER2 therapy, immunotherapy in selected settings, supportive care, and palliative interventions [2,3]. This therapeutic expansion has improved disease control for many patients, but it has also increased the complexity of care and the cumulative burden associated with treatment.

Treatment burden in advanced breast cancer is multidimensional. It includes physical toxicity, psychological distress, symptom burden, financial cost, travel, caregiver involvement, administrative work, monitoring requirements, and the amount of time that patients must devote to care. A regimen may appear clinically favorable when assessed through progression-free survival or response rate, but may be experienced as burdensome if it requires frequent hospital visits, prolonged infusions, recurrent laboratory monitoring, intensive imaging, toxicity management, and recovery periods.

In advanced disease, treatment burden must also be interpreted across time. A short period of intensive treatment may be acceptable if it produces meaningful symptom relief or disease control. Conversely, a chronically administered regimen with apparently modest toxicity may become burdensome if it requires repeated visits, ongoing monitoring, persistent low-grade symptoms, and continuous psychological attention to therapy.

### 7.2. Endocrine Therapy and CDK4/6 Inhibitors

For patients with hormone receptor-positive, HER2-negative advanced breast cancer, endocrine therapy combined with CDK4/6 inhibitors has become a major standard of care because of substantial improvements in progression-free and overall survival in multiple trials. For example, MONALEESA-2 demonstrated a significant overall survival benefit with ribociclib plus letrozole compared with placebo plus letrozole in postmenopausal patients with hormone receptor-positive, HER2-negative advanced breast cancer [59]. These advances have reshaped the therapeutic landscape by allowing many patients to receive effective oral-based therapy for prolonged periods.

From a time toxicity perspective, oral targeted regimens may reduce some forms of treatment burden compared with intravenous chemotherapy, particularly infusion time and repeated day-hospital attendance. However, oral therapy should not be equated with absence of time toxicity. CDK4/6 inhibitors require patient education, adherence, laboratory monitoring, dose adjustments, management of neutropenia, fatigue, diarrhea, hepatotoxicity, QT interval monitoring for ribociclib, and repeated clinical follow-up. The burden shifts partly from hospital-based treatment time to home-based self-management time.

Patients may appreciate the autonomy of oral therapy, but may also experience increased responsibility for adherence, symptom monitoring, and recognition of adverse events. The medication schedule, blood tests, side-effect vigilance, and fear of missing doses can create a persistent sense of being “on treatment”, even outside the clinic.

Objectively, clinicians should consider visit frequency, laboratory monitoring, pharmacy access, dose modification visits, and adverse-event contacts. Subjectively, they should explore whether patients experience the regimen as liberating, intrusive, reassuring, or cognitively burdensome. This distinction is particularly important for patients with anxiety, depression, cognitive difficulties, low health literacy, or limited caregiver support.

### 7.3. Anti-HER2 Therapy and the Route of Administration

HER2-positive advanced breast cancer has been transformed by anti-HER2 therapies, including monoclonal antibodies, tyrosine kinase inhibitors, and antibody–drug conjugates. Many patients receive prolonged anti-HER2 treatment across multiple lines, often with repeated infusions or combinations requiring regular hospital attendance. Although these therapies have substantially improved outcomes, they may generate significant time toxicity because of infusion schedules, cardiac monitoring, toxicity assessment, treatment sequencing, and supportive care needs.

The route of administration is a clinically important determinant of time burden. Intravenous monoclonal antibodies may require prolonged chair time, venous access, pharmacy preparation, observation, and nursing resources. Subcutaneous formulations can reduce administration time and may improve convenience for patients and healthcare systems. In the PHranceSCa randomized phase II study, most patients preferred the fixed-dose combination of pertuzumab and trastuzumab for subcutaneous injection over intravenous administration, with reduced clinic time and comfort during administration among the main reasons for preference [60].

Healthcare professionals also reported markedly shorter treatment-room time with the subcutaneous formulation compared with intravenous administration [60].

Although the PHranceSCa population was in the early breast cancer setting, the implications are highly relevant for advanced disease, where anti-HER2 therapy may continue for long periods. In metastatic HER2-positive breast cancer, repeated reductions in chair time and hospital attendance may accumulate into a meaningful preservation of patient time. For patients who are responding to treatment and living with metastatic disease as a chronic condition, even small reductions in visit duration may have substantial cumulative value.

Time savings should not be assessed only at the level of administration time. The broader care pathway must also be considered: travel, waiting, blood tests, cardiac monitoring, management of infusion or injection-related reactions, and coordination with other therapies. Subcutaneous therapy may reduce one component of time toxicity, but its full value depends on whether care pathways are redesigned to avoid unnecessary hospital time rather than simply shortening one step within an otherwise burdensome process.

### 7.4. Antibody–Drug Conjugates (ADC)

Antibody–drug conjugates (ADC) have become increasingly important in advanced breast cancer, including HER2-positive, HER2-low, and triple-negative disease. Trastuzumab deruxtecan significantly improved progression-free and overall survival compared with standard chemotherapy in patients with previously treated HER2-low metastatic breast cancer in DESTINY-Breast04 [61]. Sacituzumab govitecan improved progression-free and overall survival compared with single-agent chemotherapy in patients with relapsed or refractory metastatic triple-negative breast cancer in ASCENT [62]. These agents represent major therapeutic advances, but their time toxicity profile requires careful consideration.

ADC-related time toxicity arises from several sources. First, these therapies are usually administered intravenously and require repeated infusion visits. Second, they may require careful monitoring for adverse events, including nausea, fatigue, myelosuppression, diarrhea, alopecia, interstitial lung disease or pneumonitis in the case of trastuzumab deruxtecan, and neutropenia or diarrhea with sacituzumab govitecan. Third, adverse events may generate unplanned healthcare contacts, dose delays, imaging, supportive medications, emergency assessments, and recovery time. A recent systematic review and meta-analysis emphasized that ADCs in metastatic breast cancer are associated with clinically relevant adverse events that may affect quality of life [63].

The time burden of ADCs is therefore not limited to infusion duration. It includes pre-treatment evaluation, laboratory monitoring, toxicity surveillance, management of adverse events, patient education, and post-treatment recovery. In the case of trastuzumab deruxtecan, monitoring for respiratory symptoms and early recognition of interstitial lung disease are clinically essential. This may increase the cognitive and emotional burden for patients, who must remain vigilant for new symptoms and may experience anxiety about potentially serious toxicity.

From a psychiatric and psychological perspective, ADCs illustrate the central dilemma of modern oncology: highly effective treatments may also generate complex burdens. For many patients, the survival and disease-control benefits justify the treatment demands. However, time toxicity should still be discussed explicitly, especially when patients have high symptom burden, limited social support, long travel distance, previous adverse experiences with chemotherapy, or strong preferences for minimizing hospital time.

### 7.5. Chemotherapy and Recurrent Treatment Cycles

Chemotherapy remains a relevant therapeutic option in advanced breast cancer, particularly in endocrine-resistant hormone receptor-positive disease, triple-negative disease, rapidly progressive disease, visceral crisis, or later-line settings. Compared with some targeted or endocrine-based regimens, chemotherapy may be associated with more visible treatment burden because of infusion schedules, acute toxicities, cumulative adverse effects, frequent monitoring, and recovery time.

The temporal structure of chemotherapy is psychologically important. Many patients experience the treatment cycle as a repeated sequence of anticipation, infusion, acute toxicity, partial recovery, and preparation for the next cycle. This rhythm can dominate daily life, limiting spontaneity and making the patient’s future feel organized around treatment rather than personal priorities. Fatigue, nausea, neuropathy, alopecia, mucositis, cytopenias, infection risk, and emotional distress may extend the burden of each treatment well beyond the infusion day [64].

Chemotherapy-related time toxicity also includes unplanned care. Febrile neutropenia, dehydration, uncontrolled symptoms, infections, pain, gastrointestinal toxicity, and treatment complications may lead to urgent visits or hospitalizations. These episodes are particularly disruptive because they interrupt the patient’s remaining non-medical time and may reinforce feelings of vulnerability and loss of control. For caregivers, chemotherapy-related complications may require sudden availability, transportation, symptom monitoring, and emotional support.

In shared decision-making, chemotherapy should therefore be described not only in terms of response probability and adverse events, but also in terms of expected treatment rhythm. Patients may benefit from understanding how many clinic visits are expected, how long infusions may take, what monitoring is required, how much recovery time is typical, and which complications may require urgent care. Such discussions can reduce uncertainty and help align treatment choice with patient values.

### 7.6. Immunotherapy in Selected Subgroups

Immunotherapy has become relevant for selected patients with advanced triple-negative breast cancer, particularly in combination with chemotherapy in PD-L1-positive disease. KEYNOTE-355 showed that pembrolizumab plus chemotherapy improved outcomes compared with chemotherapy alone in patients with previously untreated locally recurrent inoperable or metastatic triple-negative breast cancer whose tumors expressed PD-L1 [65,66]. This has expanded therapeutic options for a subgroup of patients with aggressive disease.

From a time toxicity perspective, immunotherapy introduces specific considerations. Treatment often requires repeated intravenous administration, combination with chemotherapy, immune-related adverse event surveillance, endocrine and organ-function monitoring, and possible specialist referral for toxicity management. Immune-related adverse events may be delayed, multisystemic, and sometimes require prolonged corticosteroid treatment, hospitalization, or long-term follow-up. Therefore, even when immunotherapy is well tolerated, the need for vigilance can increase both objective and subjective burden.

Psychologically, immunotherapy may also generate a distinctive form of uncertainty. Patients may hear that immune-related toxicities can occur unpredictably and may involve multiple organs. This can increase bodily hypervigilance, especially in anxious patients. At the same time, the promise of durable response may make treatment-related time feel highly meaningful [67]. As with other treatments, the subjective value of time spent on immunotherapy depends on the patient’s expectations, perceived benefit, fear of toxicity, and experience of care.

### 7.7. Monitoring, Imaging, and the Burden of Surveillance

Treatment burden in advanced breast cancer is not generated only by anticancer drugs. Monitoring itself can produce substantial time toxicity. Patients may undergo repeated blood tests, imaging, cardiac assessments, symptom evaluations, biomarker testing, biopsy procedures, and specialist visits. These assessments are necessary for safe and effective care, but they may also fragment daily life and intensify psychological distress.

Imaging is a particularly important source of time toxicity because it combines logistical burden with emotional burden. Radiological reassessment determines whether treatment is continued, changed, or stopped. Therefore, scan intervals can structure the patient’s psychological life, creating recurrent periods of anticipatory anxiety and post-result emotional adjustment. In advanced disease, imaging is not a neutral monitoring procedure; it is a decision point that may redefine prognosis, treatment options, and the patient’s sense of future [68].

Cardiac monitoring in HER2-positive disease, laboratory monitoring during CDK4/6 inhibitor therapy, toxicity assessment during ADC treatment, and immune-related adverse event surveillance during immunotherapy all illustrate the same principle: safe treatment requires time. The clinical challenge is not to eliminate monitoring, but to avoid unnecessary duplication, poor scheduling, excessive waiting, and fragmented appointments. A patient-centered model should aim to cluster visits, coordinate tests, use local services when appropriate, and reduce avoidable healthcare contact.

### 7.8. Treatment Burden Across the Disease Trajectory

Time toxicity in advanced breast cancer should be interpreted longitudinally. At diagnosis of metastatic disease, patients may accept intensive assessment and treatment initiation because the priority is disease control and stabilization. During response or stable disease, the same intensity of contact may become more burdensome if it interferes with attempts to resume normal life. At progression, time toxicity may increase again because of additional imaging, biopsies, molecular testing, treatment changes, and emotional destabilization. In later lines, the proportionality between expected benefit and time burden becomes increasingly important.

This trajectory-based perspective is essential for clinical decision-making. The same treatment schedule may have different meanings at different points in the illness. A patient may initially value frequent contact as a form of reassurance, but later experience it as intrusive. Another patient may prefer close monitoring after progression but reduced visits during stable disease. Therefore, time toxicity should not be assessed once and assumed to remain stable. It should be revisited whenever disease status, treatment goals, symptoms, psychiatric state, or personal priorities change.

Advanced breast cancer is also characterized by repeated transitions: from first-line to subsequent-line therapy, from endocrine sensitivity to endocrine resistance, from oral therapy to intravenous treatment, from disease control to progression, and sometimes from active disease-directed therapy to palliative-focused care [1].

**Figure 3:**
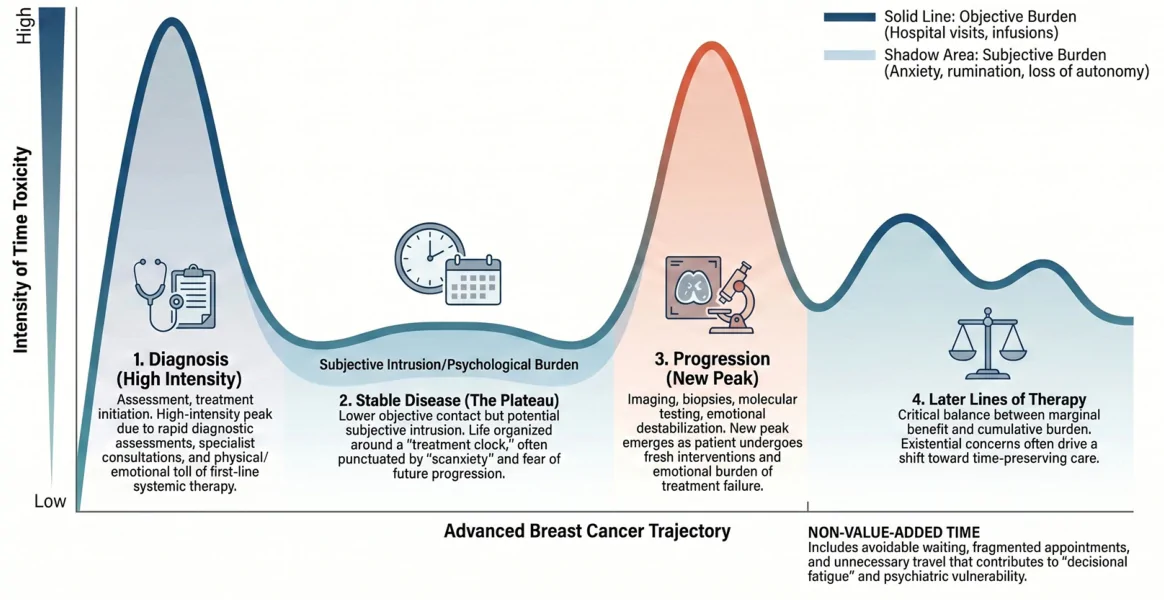
Longitudinal fluctuations of time toxicity across the advanced breast cancer trajectory. Note: This schematic tracks variation in temporal burden from initial diagnosis through subsequent lines of therapy, identifying peaks associated with diagnostic transitions and disease progression, with the orange tone specifically marking the critical peak of progression. This figure represents a conceptual illustration derived from the narrative synthesis. It does not constitute a validated clinical model or decision algorithm.

Each transition may alter the balance between benefit and burden. Integrating time toxicity into these discussions can help ensure that treatment remains goal-concordant. Time toxicity is not a static burden but a dynamic phenomenon; Figure 3 maps these longitudinal fluctuations identifying specific peaks of intensity corresponding to diagnostic transitions and disease progression.

### 7.9. Implications for Patient-Centered Treatment Selection

The treatment burden associated with advanced breast cancer cannot be evaluated solely through drug efficacy. This is particularly important when therapeutic options have comparable efficacy but differ in burden, or when expected benefit is modest.

In practice, clinicians should make time burden explicit during treatment discussions. Patients should be informed about expected treatment schedule, monitoring requirements, typical visit duration, probability of unplanned care, recovery time, and potential impact on daily life [4]. These considerations provide the basis for incorporating time burden into shared decision-making.

## 8. Integrating Time Toxicity into Shared Decision-Making

### 8.1. Time Toxicity as a Decision-Relevant Outcome

Shared decision-making is particularly important in advanced breast cancer because treatment choices are often preference-sensitive. Patients and clinicians may need to compare options that differ not only in expected efficacy, but also in toxicity, route of administration, monitoring requirements, probability of unplanned healthcare use, financial implications, psychological burden, and impact on daily life. The principles of preference elicitation and shared decision-making are supported by broad oncology and palliative care literature. Their application to time toxicity in advanced breast cancer is clinically relevant but has not yet been prospectively tested as a distinct decision-support intervention.

Traditional oncological conversations often prioritize survival outcomes, tumor response, and adverse events. A patient may reasonably ask how much time the treatment will require, how often hospital attendance will be necessary, and whether the expected benefit justifies the time spent in care.

This perspective is consistent with contemporary oncology communication guidance, which emphasizes the importance of discussing treatment goals, prognosis, patient values, and care preferences in a clear and empathic manner [69,70]. Time toxicity provides a concrete language through which these values can be translated into clinical discussion. It allows clinicians to move beyond abstract statements about quality of life and toward specific questions about how treatment will reorganize the patient’s calendar, emotional life, family roles, and sense of autonomy.

### 8.2. Eliciting Patient Values and Preferences

A central step in integrating time toxicity into shared decision-making is the explicit elicitation of patient values. Patient preferences in breast cancer are heterogeneous and may include survival prolongation, avoidance of severe adverse events, preservation of quality of life, reduction in hospital visits, maintenance of autonomy, ability to work, family responsibilities, body image, sexual functioning, and emotional stability. Recent preference research confirms that patients with breast cancer may value not only efficacy, but also treatment burden, side-effect profile, convenience, and the preservation of daily functioning [71,72].

In advanced disease, values may change across the illness trajectory. At the start of metastatic treatment, some patients may prioritize maximal disease control and accept high time burden. During prolonged stable disease, the same patient may become more concerned with preserving daily life and reducing hospital attendance. At progression, priorities may shift again, depending on symptom burden, available treatment options, family circumstances, prognosis awareness, and psychological adaptation. Therefore, patient preferences should not be assessed once and assumed to remain stable. They should be revisited at each major transition.

Clinicians may ask how much time in hospital feels acceptable, which activities the patient most wishes to preserve, and whether the expected benefit appears proportionate to the time required by treatment.

Such conversations are particularly important because patients may not spontaneously raise time-related concerns. Some may fear being perceived as ungrateful, non-adherent, or insufficiently motivated. Others may assume that hospital time is unavoidable and therefore not discussable. A clinician who proactively asks about time burden legitimizes the patient’s experience and creates space for a more realistic discussion of treatment value.

### 8.3. Balancing Survival Benefit, Time Burden, and Psychological Costs

Shared decision-making in advanced breast cancer often involves trade-offs. A treatment may offer modest survival benefit but require frequent hospital attendance, substantial monitoring, and a high probability of adverse events. Another option may provide less intensive disease control but better preservation of time at home and daily functioning. In such situations, there is no universally correct decision. The appropriate choice depends on clinical evidence, prognosis, treatment goals, and the patient’s subjective valuation of time.

Time toxicity is especially relevant when treatments provide marginal or uncertain benefit. Patients may differ substantially in how they value a small survival gain relative to treatment burden. Some may accept intensive therapy for even a limited chance of benefit, while others may prioritize fewer hospital visits, reduced toxicity, and more predictable personal time. Research on cancer treatment preferences suggests that patients often trade survival gains for lower burden, fewer adverse effects, and better quality of life, although preferences vary considerably across individuals [72].

### 8.4. Communication About Prognosis and Time

Discussion of time toxicity inevitably intersects with communication about prognosis. In advanced breast cancer, the meaning of time burden depends partly on the patient’s understanding of disease trajectory, expected benefit, and realistic treatment goals. If prognosis is not discussed clearly, patients may overestimate the benefit of therapy and underestimate the opportunity cost of time spent in treatment. Conversely, overly abrupt or poorly supported prognostic communication may increase distress, hopelessness, or disengagement from care.

High-quality patient–clinician communication requires honesty, empathy, and adaptation to the patient’s informational preferences. ASCO Patient-Clinician Communication guidelines emphasize that clinicians should provide information about prognosis and treatment options in a manner that supports patient understanding, values-based decision-making, and emotional adjustment [69,70]. In the context of time toxicity, this means helping patients understand not only how long a treatment may extend survival or control disease, but also what kind of time the treatment is likely to produce.

The discussion should include the expected frequency of visits and the anticipated duration of each treatment day. Clinicians should also explain the likely recovery time, the possibility of hospitalization, and the need for imaging or laboratory monitoring. Patients should be informed about when treatment benefit can reasonably be assessed and whether treatment may improve, preserve, or worsen symptoms. These practical details may make prognostic information more meaningful because they translate abstract survival estimates into lived experience.

Psychiatric and psychological expertise can support these conversations by helping clinicians recognize when emotional distress interferes with comprehension, when hope and realism are in tension, and when a patient’s preference may reflect untreated depression, anxiety, demoralization, or cognitive overload. Importantly, integrating time toxicity into prognostic communication does not mean discouraging active treatment. Rather, it means helping patients make informed choices about how treatment-related time fits within their values and goals.

### 8.5. The Role of Decision Aids and Structured Tools

Decision aids may help incorporate time toxicity into shared decision-making by presenting information about treatment options in a structured, understandable, and preference-sensitive manner. In breast cancer, decision aids have been developed for several treatment contexts, and recent reviews suggest that they can support patient involvement, improve knowledge, and clarify preferences [73]. However, many existing tools still focus primarily on efficacy, adverse events, and general quality of life, while less systematically addressing time burden.

A time toxicity-informed decision aid for advanced breast cancer should include several domains: expected treatment schedule, mode of administration, visit frequency, monitoring requirements, estimated time in clinic, travel burden, risk of unplanned healthcare contact, recovery time, caregiver involvement, and possible psychological impact. It should also include value-clarification exercises, allowing patients to indicate how they weigh survival benefit, symptom control, time at home, autonomy, treatment convenience, emotional reassurance, and avoidance of hospital settings.

Such tools may be particularly helpful for patients who feel overwhelmed by complex treatment information. Decision aids can reduce cognitive burden by organizing options visually and explicitly highlighting trade-offs. However, they should not replace clinician communication. Instead, they should support a relational process in which the clinician helps the patient interpret evidence in light of their values, prognosis, psychiatric vulnerability, and family context.

A limitation of decision aids is that they may unintentionally increase decisional burden if too much information is provided without adequate guidance. Therefore, tools should be designed with attention to health literacy, emotional state, cultural background, and cognitive capacity.

### 8.6. Family, Caregivers, and Relational Decision-Making

Advanced breast cancer decisions are rarely made in isolation. Partners, children, relatives, and informal caregivers often influence treatment choices, provide transportation, manage schedules, accompany patients to visits, monitor symptoms, and support medication adherence. Because caregiver time is part of the broader time toxicity of cancer care, shared decision-making should include the relational context whenever the patient wishes to involve others.

Caregiver involvement may be beneficial, but it can also create tension. Family members may prioritize survival prolongation more strongly than the patient, or may encourage continued treatment even when the patient is increasingly burdened. Conversely, caregivers may experience exhaustion and silently hope for less intensive care while feeling guilty about expressing this. The patient may continue treatment partly to protect family members from the feeling that “nothing more is being done”. These relational dynamics can shape how time toxicity is discussed and valued [74]. A psychiatry- and psychology-informed approach should explore not only the patient’s individual preferences, but also the interpersonal pressures surrounding treatment decisions. Clinicians may ask whether the patient feels free to express their priorities, whether family expectations influence their decisions, whether caregiver burden is becoming unsustainable, and whether discussions about prognosis and goals of care have occurred within the family. These questions are essential because time toxicity is often distributed across the family system.

When caregivers are involved, clinicians should clarify that the patient’s values remain central. Family input can enrich the discussion, but should not replace the patient’s voice. In advanced breast cancer, protecting patient autonomy includes protecting the patient’s right to define what constitutes meaningful time.

### 8.7. Psychiatric Consultation Within Shared Decision-Making

Psychiatric and psychological consultation can play an important role when time toxicity becomes central to treatment decisions. This is particularly relevant when the patient appears overwhelmed, ambivalent, demoralized, highly anxious, cognitively overloaded, or when clinicians are uncertain whether a preference for less burdensome care reflects autonomous values or treatable psychiatric symptoms.

A psychiatric assessment can help distinguish between several clinically different situations. A patient may refuse a burdensome treatment because of untreated major depression and hopelessness; another may decline the same treatment after a clear and values-consistent appraisal of expected benefit and burden. A patient may request intensive monitoring because of uncontrolled anxiety; another may request it because frequent contact genuinely provides reassurance and aligns with their coping style. These distinctions matter because they lead to different interventions.

The role of psychiatry is not to “correct” patient preferences toward more treatment or less treatment. Rather, it is to support decisional capacity, reduce modifiable suffering, clarify values, and help ensure that choices are not primarily driven by untreated psychiatric symptoms, coercive family dynamics, misinformation, or unbearable anxiety. When depression, panic, insomnia, trauma-related symptoms, or demoralization are treated, patients may become better able to engage in shared decision-making and to articulate how they wish to use their time [10].

Psychological interventions may also help patients tolerate uncertainty, manage scanxiety, communicate with family, and identify activities that make time meaningful. In this way, psycho-oncology contributes not only to symptom reduction, but also to the ethical quality of decision-making.

### 8.8. Toward Time-Concordant Care

The integration of time toxicity into shared decision-making leads to the broader concept of time-concordant care. Time-concordant care can be defined as care in which the amount, structure, and setting of healthcare-related time are aligned with the patient’s clinical needs, prognosis, psychological state, relational context, and personal values. This concept extends goal-concordant care by emphasizing that the way time is used is itself a central component of patient-centered oncology [75].

In practical terms, time-concordant care requires clinicians to discuss expected benefit and time burden together. It also requires healthcare systems to provide realistic information about visit frequency, waiting times, travel requirements, monitoring schedules, and available alternatives such as telemedicine, local laboratory testing, subcutaneous formulations, oral therapies, or clustered appointments. Without system-level support, clinicians may recognize time toxicity but remain unable to reduce it.

Time-concordant care is especially relevant in advanced breast cancer because patients may live for years with metastatic disease while undergoing sequential treatment. The goal is not always to minimize healthcare contact. Some patients may prefer frequent contact because it provides reassurance, symptom control, and relational continuity with the care team. Rather, the goal is to ensure that healthcare-related time is meaningful, proportionate, and aligned with what the patient values.

**Figure 4:**
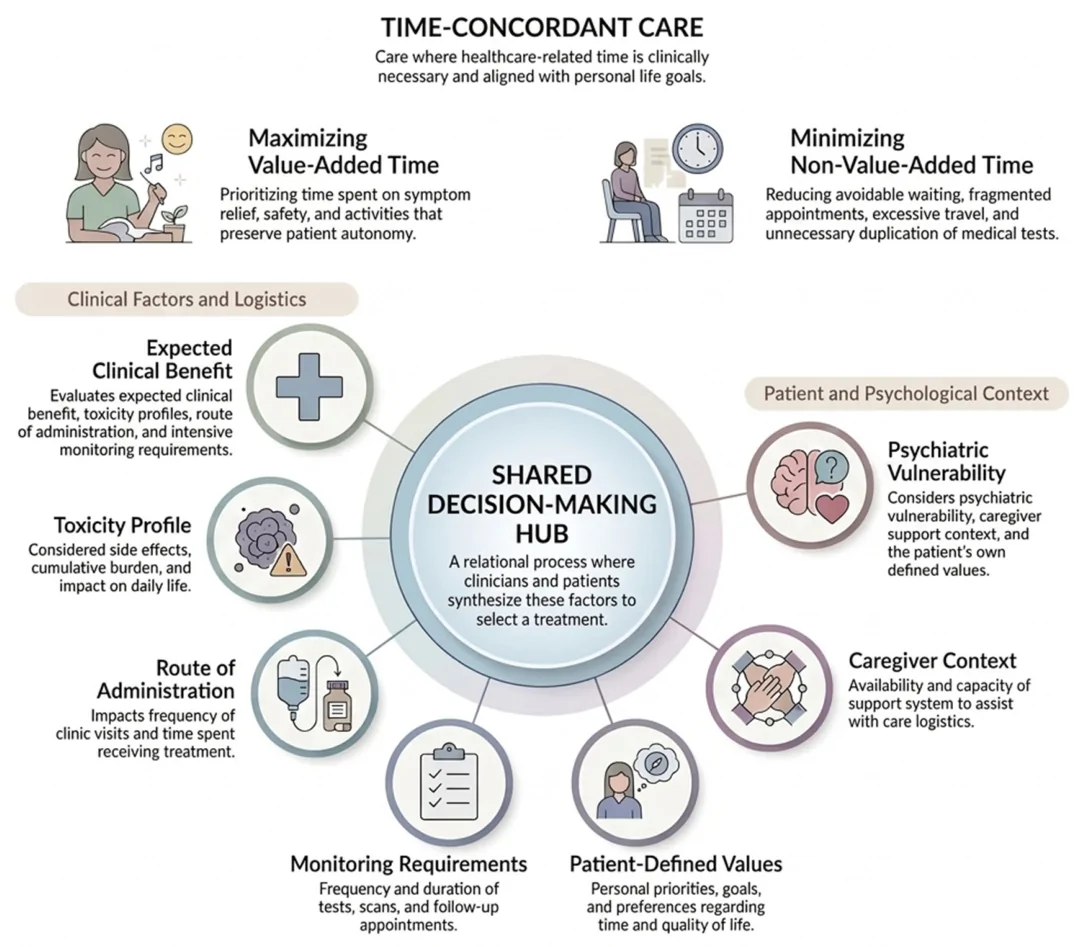
Integration of time toxicity into shared decision-making. Note: Time toxicity should be incorporated into treatment discussions together with expected clinical benefit, toxicity profile, route of administration, monitoring requirements, psychiatric vulnerability, caregiver context, and patient-defined values. This approach supports time-concordant care, in which healthcare-related time is proportionate, meaningful, clinically necessary, and aligned with the patient’s goals. This figure represents a conceptual illustration derived from the narrative synthesis. It does not constitute a validated clinical model or decision algorithm.

Thus, incorporating time toxicity into shared decision-making shifts the clinical question from “What treatment can we offer next?” to “Which treatment offers the most meaningful balance between benefit, burden, and the patient’s use of time?”. This shift is essential for a psychiatry- and psychology-informed model of advanced breast cancer care. A practical model for integrating time toxicity into shared decision-making is shown in Figure 4. The model emphasizes that treatment selection should be based not only on expected oncological benefit, but also on temporal burden, psychological vulnerability, caregiver context, and the patient’s own valuation of time.

## 9. Reducing Time Toxicity: Clinical, Organizational, and Psychological Strategies

### 9.1. From Measurement to Intervention

Recognizing time toxicity is clinically useful only if it leads to strategies capable of reducing avoidable burden while preserving the quality, safety, and effectiveness of care. In advanced breast cancer, the aim should not be to minimize healthcare contact indiscriminately, because some visits are essential for treatment delivery, symptom control, monitoring, emotional reassurance, and therapeutic alliance. Rather, the goal is to distinguish between value-added and non-value-added time. Value-added time refers to healthcare-related time that contributes meaningfully to survival, symptom relief, safety, psychological support, or patient-defined goals. Non-value-added time includes avoidable waiting, fragmented appointments, unnecessary duplication of tests, poorly coordinated visits, preventable emergency department use, excessive travel, and administrative inefficiencies [58].

Reducing time toxicity therefore requires interventions at multiple levels: treatment selection, route of administration, care pathway design, digital monitoring, psycho-oncological support, caregiver involvement, and health system organization. In advanced breast cancer, these interventions should be individualized according to disease subtype, line of therapy, prognosis, treatment goals, toxicity profile, psychiatric vulnerability, social support, distance from care, and patient preferences. A patient-centered approach should ask not only whether a treatment can be delivered, but whether it can be delivered in a way that preserves meaningful time.

Importantly, strategies to reduce time toxicity should not be interpreted as de-escalation by default. In some cases, reducing unnecessary time burden may allow patients to remain on effective therapy longer, improve adherence, decrease distress, and preserve functioning. In other cases, it may support a transition toward less intensive or more comfort-focused care when the expected benefit of further treatment no longer justifies the temporal, physical, and psychological cost.

### 9.2. Treatment Optimization and Route of Administration

One of the most direct ways to reduce time toxicity is to select treatments and administration modalities that minimize unnecessary healthcare contact without compromising efficacy. In advanced breast cancer, this includes consideration of oral therapies, subcutaneous formulations, shorter infusion protocols when clinically appropriate, home-based supportive care, and rationalized monitoring schedules.

Oral endocrine therapy and oral targeted agents, including CDK4/6 inhibitors, may reduce infusion-related time and day-hospital attendance compared with intravenous chemotherapy. However, as discussed above, oral therapy does not eliminate treatment burden; it shifts part of the burden to home-based adherence, laboratory monitoring, toxicity recognition, and self-management. Therefore, oral regimens should be accompanied by clear education, symptom reporting pathways, and psychological support when needed.

Subcutaneous formulations may reduce chair time, preparation time, and treatment-room occupancy. In HER2-positive breast cancer, the fixed-dose subcutaneous combination of pertuzumab and trastuzumab was preferred by most patients in the PHranceSCa study, with reduced clinic time and comfort during administration among the most frequently reported reasons for preference [60]. Although this trial was conducted in early breast cancer, the implications are particularly relevant for advanced disease, where repeated administration over long periods may lead to substantial cumulative time savings.

Treatment optimization also requires attention to proportionality. When several regimens are clinically reasonable, time burden should be discussed alongside efficacy, toxicity, route of administration, monitoring intensity, and patient values. For example, a regimen with slightly greater efficacy but substantially higher time burden may be appropriate for one patient and unacceptable for another. Conversely, a patient may willingly accept time-intensive therapy if it offers meaningful symptom control, disease response, or psychological reassurance.

### 9.3. Redesigning Care Pathways

Many components of time toxicity arise not from the anticancer treatment itself, but from the organization of care. Fragmented scheduling, repeated visits on different days, long waiting times, poorly coordinated laboratory and imaging appointments, and unclear communication can substantially increase the patient’s time burden. In advanced breast cancer, where patients may require prolonged and repeated interaction with oncology services, small inefficiencies can accumulate into a major loss of time.

Care pathway redesign should aim to cluster appointments whenever possible. Blood tests, oncology consultations, treatment administration, supportive care visits, imaging, pharmacy access, and psychosocial assessment should be coordinated to reduce multiple separate trips. One-stop or same-day models may be particularly valuable for patients living far from the cancer center, those with limited mobility, those dependent on caregivers for transport, and those with high anxiety related to medical visits.

Reducing waiting time is also essential. Waiting is not only an operational inconvenience; it is psychologically charged, especially when patients are awaiting scan results, treatment decisions, or confirmation of progression. Clear scheduling, predictable appointment flows, timely communication of results, and proactive management of delays may reduce both objective and subjective time toxicity. In this sense, administrative efficiency becomes a form of psychological care [76].

Care coordination is particularly important during transitions: initiation of a new treatment line, progression, toxicity-related dose modification, hospitalization, and transition to palliative-focused care. These phases often generate additional appointments, uncertainty, and emotional distress. A coordinated pathway can reduce avoidable contacts and help patients understand what to expect, thereby preserving both time and perceived control.

### 9.4. Telemedicine and Hybrid Models of Care

Telemedicine has the potential to reduce travel time, waiting-room exposure, caregiver burden, and disruption of daily life. For patients with advanced breast cancer, remote consultations may be useful for selected follow-up visits, toxicity checks, review of stable symptoms, psychological support, survivorship-style discussions during prolonged disease control, and caregiver meetings. Telemedicine may be especially beneficial for patients living far from oncology centers, those with mobility limitations, those with high fatigue, and those who experience hospital visits as psychologically distressing.

Telemedicine should not be assumed to reduce time toxicity automatically. Recent evidence in advanced cancer suggests that telehealth may reduce some forms of in-person contact while adding other forms of healthcare interaction, and that overall healthcare contact days may not necessarily decrease [56]. Remote care may also shift administrative and monitoring responsibilities to patients and caregivers. For example, patients may need to coordinate local blood tests, manage digital platforms, report symptoms electronically, obtain medications, or arrange additional in-person assessments if concerns arise.

Hybrid care models should be designed carefully. Telemedicine is most likely to reduce time toxicity when it replaces a visit that does not require physical examination, infusion, imaging, or urgent assessment. Its effectiveness also depends on integration with local services, accessible digital platforms, and adequate technological access and health literacy. Conversely, telemedicine may increase burden if it creates duplicated visits, unclear responsibilities, or anxiety due to perceived distance from the care team.

Patient preference is critical. Some patients may feel safer and more connected with in-person visits, while others may experience hospital attendance as exhausting and intrusive. A time toxicity-informed approach should therefore offer telemedicine selectively, according to clinical appropriateness and patient values.

### 9.5. Digital Symptom Monitoring and Early Toxicity Management

Digital symptom monitoring may reduce time toxicity by identifying adverse events early, preventing avoidable emergency department visits, supporting timely dose adjustments, and improving communication between patients and oncology teams. Electronic patient-reported outcome systems are particularly relevant because many symptoms are under-recognized during routine care. In a randomized trial of patients receiving routine cancer treatment, electronic symptom monitoring using patient-reported outcomes was associated with improved clinical outcomes, including longer overall survival in extended follow-up [77].

In advanced breast cancer, digital symptom monitoring could be particularly useful for patients receiving therapies associated with fatigue, diarrhea, neutropenia, nausea, pain, neuropathy, endocrine symptoms, respiratory symptoms, or immune-related adverse events. Timely detection may allow early intervention and reduce unplanned healthcare use. For patients receiving antibody–drug conjugates, for example, structured symptom reporting may support earlier recognition of respiratory symptoms suggestive of interstitial lung disease or clinically relevant gastrointestinal and hematological toxicity. For patients receiving CDK4/6 inhibitors, remote monitoring may support adherence, laboratory coordination, and early management of adverse events.

Digital monitoring must be implemented without increasing patient burden. Excessive questionnaires, frequent alerts, unclear response pathways, or poorly integrated platforms may create a new form of digital time toxicity. Digital tools should be brief, clinically actionable, integrated into workflow, and accompanied by clear instructions about when and how the care team will respond. The aim is not to medicalize the patient’s home further, but to prevent avoidable escalation and preserve time outside healthcare settings.

### 9.6. Early Palliative Care as Time-Preserving Care

Early palliative care can contribute to reducing time toxicity by improving symptom management, clarifying goals of care, supporting communication, reducing crisis-driven healthcare use, and helping patients align treatment decisions with personal priorities. In advanced cancer, palliative care should not be understood as the opposite of active treatment, but as an integrated layer of care focused on quality of life, symptom relief, psychosocial support, and decision-making.

The landmark randomized trial by Temel et al. showed that early palliative care in metastatic non-small-cell lung cancer improved quality of life and mood, and was associated with less aggressive care near the end of life [78]. A Cochrane review also concluded that early palliative care may improve quality of life and symptom intensity in advanced cancer, although effects vary across studies and settings [79]. Although much of this evidence is not breast-cancer-specific, its principles are highly applicable to advanced breast cancer, especially when patients face recurrent treatment decisions, cumulative symptoms, and increasing uncertainty.

From a time toxicity perspective, early palliative care may help distinguish between treatments that preserve meaningful time and those that consume time without proportional benefit. It may also reduce avoidable emergency visits by improving anticipatory symptom management and advance care planning. Psychologically, early palliative care can support patients in articulating what they want their time to be used for, which activities matter most, and how treatment should be adapted as illness evolves.

For patients with advanced breast cancer, early palliative care should be introduced not only when disease-directed options are exhausted, but when symptom burden, emotional distress, treatment complexity, or decisional conflict become substantial. This approach can help preserve time as a lived resource rather than merely as a survival metric.

### 9.7. Psycho-Oncological and Psychiatric Interventions

Psychological and psychiatric interventions may address several subjective components associated with time toxicity, although their direct effect on time toxicity as a distinct outcome has not been established. Evidence supporting these interventions derives mainly from general psycho-oncology and advanced cancer populations rather than from trials specifically designed around time toxicity in advanced breast cancer.

Even when the number of appointments cannot be reduced, psychological care may decrease anticipatory anxiety, scan-related distress, demoralization, insomnia, depressive symptoms, decisional fatigue, and perceived loss of control. In this sense, psycho-oncology can reduce the psychological toxicity of time.

Interventions should be selected according to the patient’s needs. Psychoeducation may help patients understand treatment schedules, anticipate side effects, prepare for scans, and distinguish expected symptoms from warning signs. Cognitive-behavioral approaches may reduce catastrophic thinking, avoidance, insomnia, and anxiety around medical visits. Supportive-expressive therapy may help patients process fear, grief, relational concerns, and role changes. Meaning-centered psychotherapy may be particularly relevant in advanced disease, because it focuses on meaning, purpose, dignity, legacy, and the preservation of identity despite life-limiting illness. Individual meaning-centered psychotherapy has shown benefits for psychological and existential distress in patients with advanced cancer [80].

Psychiatric treatment is also important when time toxicity is amplified by major depression, anxiety disorders, panic symptoms, trauma-related symptoms, severe insomnia, or demoralization. Pharmacological treatment, when clinically indicated, may improve mood, sleep, energy, anxiety, and the capacity to engage in decision-making. Psychiatric consultation can also help evaluate decisional capacity, distinguish autonomous preference from treatable psychopathology, and support communication between patient, family, and oncology team.

Psycho-oncological interventions should be integrated into the care pathway rather than offered only after crisis. Screening for distress, depression, anxiety, fear of progression, insomnia, and demoralization should occur at diagnosis of advanced disease, treatment change, progression, hospitalization, and transition points. Timely intervention may reduce the subjective intensity of time toxicity and improve the patient’s ability to use non-medical time meaningfully.

### 9.8. Caregiver Support and Relational Time

Caregiver support may reduce time toxicity by improving coordination, preventing crisis-driven visits, supporting symptom monitoring, and reducing patient anxiety. However, caregivers also need protection from overload. If the care pathway assumes unlimited caregiver availability, time toxicity is simply transferred from the healthcare system to the family. This may increase caregiver distress, financial strain, relational tension, and patient guilt [81].

Clinicians should ask explicitly about caregiver time, availability, work responsibilities, emotional strain, transportation capacity, and need for support. When possible, appointments should be scheduled with caregiver constraints in mind. Telemedicine may help caregivers participate without travel, but may also add responsibilities if not well coordinated. Social work, nursing navigation, psychological support, and community resources can help reduce the hidden burden on families.

Relational time is also important. For many patients with advanced breast cancer, time with family, children, partners, friends, and meaningful relationships is among the most valued dimensions of life. Treatments that preserve this relational time may have value beyond what is captured by survival metrics. Conversely, treatments that repeatedly disrupt relational life may be experienced as disproportionately burdensome, even when medically reasonable.

### 9.9. Equity-Oriented Strategies

Time toxicity is not distributed equally. Patients living in rural areas, those with limited income, poor transportation, low health literacy, immigration-related barriers, disability, single parenthood, precarious employment, or limited caregiver support may experience a greater temporal burden from the same treatment schedule. Time toxicity therefore has an equity dimension.

Equity-oriented strategies include decentralized laboratory testing, coordination with local hospitals, transportation support, flexible scheduling, telemedicine when appropriate, navigation services, culturally sensitive communication, and attention to work and caregiving responsibilities. For patients with psychiatric vulnerability, equity also includes access to psycho-oncology and psychiatry, because untreated distress can magnify the subjective burden of care [82].

In advanced breast cancer, reducing time toxicity should not become a privilege available only to patients with high resources, digital literacy, or proximity to academic centers. Health systems should actively identify patients at higher risk of disproportionate time burden and adapt care pathways accordingly. This is particularly important because high time toxicity may reduce adherence, increase distress, and make effective treatment less accessible.

### 9.10. Toward a Multilevel Strategy

No single intervention can eliminate time toxicity. Effective reduction requires a multilevel strategy combining clinical judgment, patient preference elicitation, organizational redesign, digital support, psycho-oncological care, caregiver support, and equity-oriented planning. In advanced breast cancer, a multilevel strategy may begin with the selection of less time-intensive regimens and the use of oral or subcutaneous options when clinically appropriate. Organizational measures may include clustering appointments, reducing avoidable waiting, offering telemedicine selectively, and implementing digital symptom monitoring. This approach should also integrate early palliative care, psychological assessment, caregiver support, and repeated evaluation of patient priorities across the disease trajectory.

The central principle is proportionality. Healthcare-related time should be proportionate to expected clinical benefit, symptom relief, safety, and patient-defined goals. When time burden is high, clinicians should ask whether it is medically necessary, whether it can be reduced, whether it is psychologically tolerable, and whether the patient considers it worthwhile. This proportionality-based approach can help transform time toxicity from an invisible burden into a modifiable component of patient-centered care [23].

**Figure 5:**
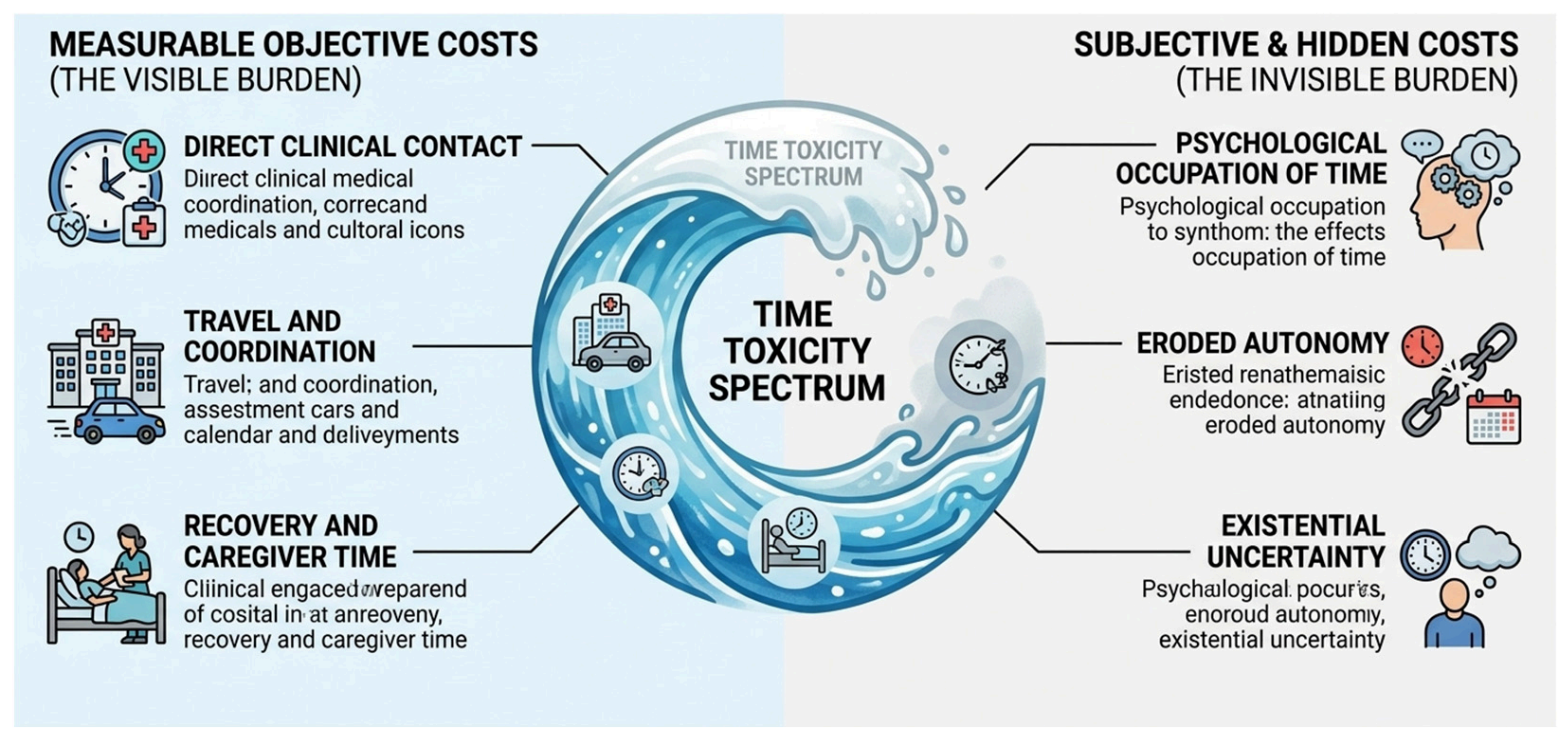
A multilevel strategy for reducing time toxicity in oncology. Note: This schematic overview integrates clinical (e.g., treatment optimization, route of administration), organizational (e.g., care pathway redesign, appointment clustering), and psychological strategies (e.g., distress screening, psycho-oncological support). The goal of these intersecting interventions is to maximize value-added time while minimizing the non-value-added burden on patients and caregivers. This figure represents a conceptual illustration derived from the narrative synthesis. It does not constitute a validated clinical model or decision algorithm.

For patients with advanced breast cancer, reducing time toxicity is not simply a matter of convenience. It is a way of preserving autonomy, dignity, relational life, psychological well-being, and the subjective value of time. In this sense, time-preserving care should be considered an essential dimension of modern oncology. Time toxicity is potentially modifiable through interventions targeting treatment selection, route of administration, care organization, digital monitoring, psycho-oncological support, caregiver involvement, and health equity. An integrated multilevel approach for reducing avoidable time toxicity and promoting time-concordant care is presented in Figure 5.

Potential strategies to reduce time toxicity in advanced breast cancer are summarized in Table 3, according to their clinical rationale, expected psychological benefit, and implementation challenges.

## 10. A Five-Dimensional Conceptual Model of Time Toxicity in Advanced Breast Cancer

### 10.1. Rationale for an Integrated Framework

The growing literature on time toxicity has made an essential contribution by drawing attention to the temporal burden of cancer care. However, most current approaches still emphasize measurable healthcare utilization, such as contact days, hospital days, clinic visits, treatment time, travel, and emergency department use. These indicators are clinically valuable, but they capture only part of the patient’s experience. In advanced breast cancer, time toxicity is not only a matter of how many days are spent in contact with the healthcare system. It also concerns how those days are experienced, how they affect emotional functioning, how they reshape family life, and how they influence the perceived meaning of the time that remains.

A psychiatry- and psychology-informed framework is therefore needed to integrate objective and subjective dimensions of time toxicity. Such a framework should not replace existing metrics, but expand them.

The proposed model conceptualizes time toxicity as a multidimensional construct composed of five interrelated domains: objective time burden, subjective time burden, psychological burden, relational and caregiver burden, and existential value of time. These domains are grounded in current conceptual analyses of time toxicity, which identify temporal burden, disruption of daily life, cumulative effect, opportunity cost, and emotional strain as defining attributes of the construct [15]. They also reflect emerging qualitative evidence showing that time commitments in advanced cancer are experienced by patients and caregivers as organizational, emotional, social, and existential burdens, not merely as healthcare utilization events [83]. The five-dimensional model proposed represents a conceptual organization of the themes identified across the reviewed literature. It is intended to distinguish the measurable temporal demands of cancer care from the subjective, psychological, relational, and existential factors that may shape their impact. The model has not been prospectively tested, validated against clinical outcomes, or developed through formal expert consensus. Its purpose is therefore heuristic and hypothesis-generating: it may support the formulation of research questions and the future development of standardized measures, but it should not currently be used as a prescriptive clinical algorithm.

**Figure 6:**
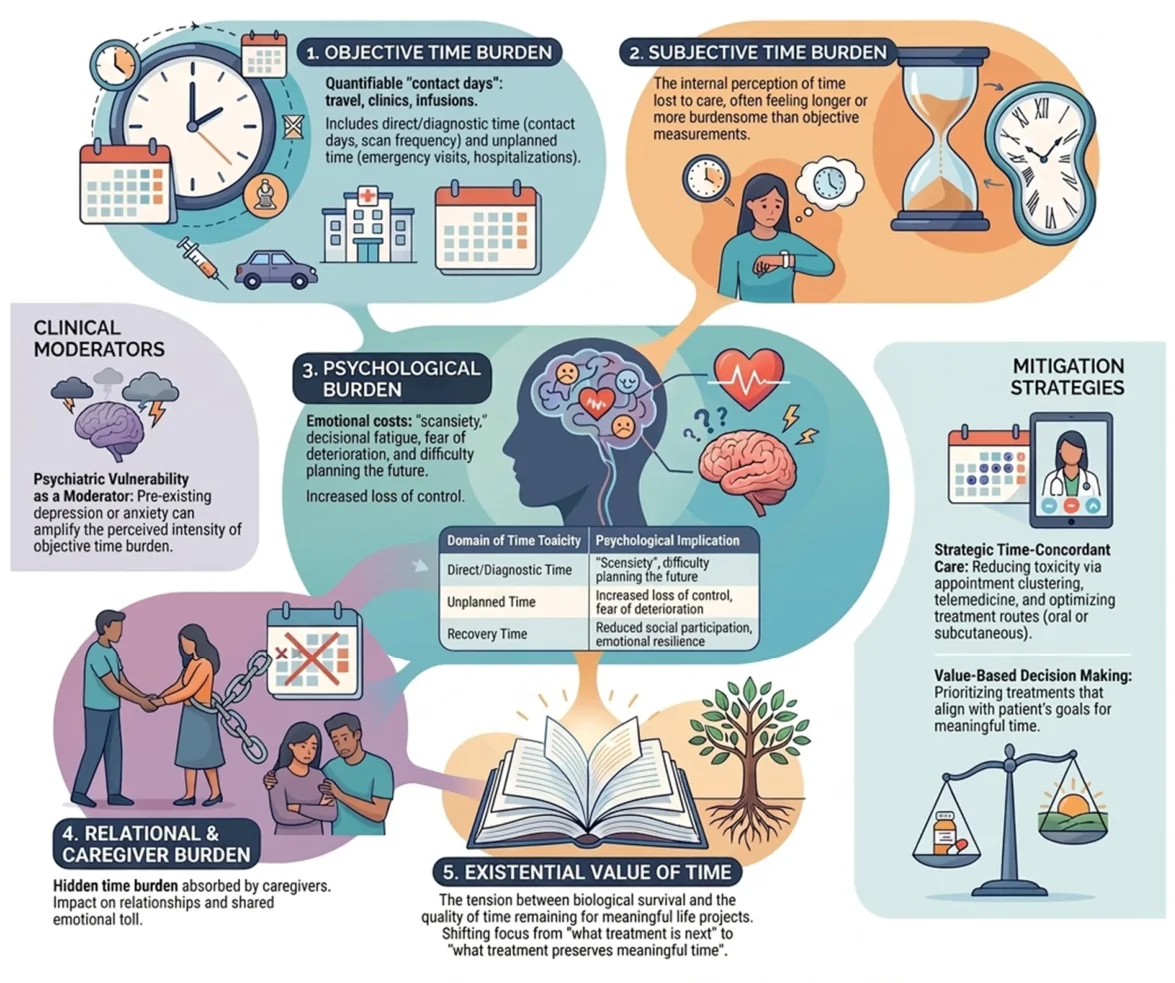
Five clinically relevant domains of time burden in advanced breast cancer care. Note: This figure summarizes five clinically relevant domains of time burden discussed in the text; it is intended as an organizing framework for future research and clinical discussion.

Figure 6 summarizes five domains that repeatedly emerge from the literature: objective healthcare-related time, subjective time burden, psychological and psychiatric modifiers, caregiver and relational burden, and the existential value of time. We use this framework only to structure the narrative and to highlight clinically relevant questions; it is not presented as a diagnostic tool or scoring system.

### 10.2. Dimension 1: Objective Time Burden

The first dimension is objective time burden. This refers to measurable time directly or indirectly consumed by cancer care. It includes treatment administration, monitoring, healthcare contacts, travel, emergency care, rehabilitation, and supportive care [18].

A single contact day can represent very different experiences: a short blood test, a prolonged infusion, a scan associated with intense anxiety, or an emergency department visit followed by admission. Objective measures should distinguish planned from unplanned care and consider both frequency and intensity.

### 10.3. Dimension 2: Subjective Time Burden

The second dimension is subjective time burden. This refers to the patient’s perception of time lost, disrupted, fragmented, or controlled by the disease and its treatment. Subjective time burden may be high even when objective healthcare contact is relatively limited. Conversely, some patients may tolerate frequent visits if they perceive them as meaningful, reassuring, or necessary.

Subjective time burden includes the feeling that life is organized around treatment cycles, scans, blood tests, appointments, and waiting for results. It also includes loss of spontaneity, reduced ability to plan, frustration with delays, perceived dependency on the healthcare system, and the sense that medical care occupies mental space even outside the clinic. Recent qualitative work has emphasized that time toxicity may disrupt daily life order and aggravate psychological distress in patients with advanced cancer [20]. This suggests that subjective time burden is not a secondary consequence of objective time use, but a core component of the construct.

Subjective time burden can be explored through direct questions about how much of the patient’s life feels organized around cancer care. Clinicians may ask whether appointments provide reassurance or create a sense of constraint and how difficult it has become to plan personal time. Recovery after visits and the perceived value of the time spent in care should also be discussed. These questions help clinicians identify burdens that are invisible in administrative data.

### 10.4. Dimension 3: Psychological Burden

The third dimension is psychological burden. This domain includes distress, anxiety, depression, demoralization, fear of progression, scanxiety, decisional fatigue, cognitive overload, insomnia, irritability, and perceived loss of control. Psychological burden is not simply a consequence of time toxicity; it can also amplify it.

Patients with pre-existing depression, anxiety, trauma history, limited coping resources, or demoralization may experience the same objective time burden as more intrusive, threatening, or exhausting.

This domain is particularly relevant from a psychiatric perspective. Cancer-related distress has been recognized as a major clinical issue across the cancer trajectory, and guidelines recommend routine screening and management [8].

Whenever possible, psychological burden should be assessed using validated patient-reported measures together with clinical evaluation.

### 10.5. Dimension 4: Relational and Caregiver Burden

The fourth dimension is relational and caregiver burden. Caregivers contribute substantially through transportation, care coordination, symptom monitoring, and medication management. Their time is part of the total temporal cost of care.

Relational burden also includes the loss of shared time. Patients with advanced breast cancer may experience distress when treatment schedules reduce time with children, partners, friends, or meaningful social roles. Caregivers may simultaneously feel committed to supporting treatment and overwhelmed by the cumulative demands of care. These dynamics may shape treatment preferences, sometimes explicitly and sometimes silently.

Assessment should consider caregiver availability, family responsibilities, work disruption, and sustainability of care [8].

### 10.6. Dimension 5: Existential Value of Time

The fifth dimension is the existential value of time. In advanced breast cancer, time is not merely a resource to be allocated efficiently. It is a finite, emotionally charged, and meaning-laden dimension of life. Patients may ask not only how long they will live, but what kind of time treatment will allow them to have.

The existential value of time includes meaning, dignity, hope, legacy, relational presence, life completion, unfinished projects, spiritual concerns, and the patient’s sense of what remains worth preserving [27,28].

This domain has direct implications for clinical decision-making. A treatment may be considered valuable if it enables the patient to reach a meaningful family event, remain active in a valued role, travel, reduce symptoms, or preserve relational time. Conversely, a treatment may be experienced as excessively burdensome if it consumes time without producing a benefit that the patient considers meaningful. In this sense, the existential value of time cannot be inferred from survival statistics alone.

Psychological interventions such as meaning-centered psychotherapy, dignity-oriented care, supportive-expressive therapy, and early palliative care can help patients clarify what makes time meaningful and how treatment decisions should reflect those priorities. The aim is not to oppose survival prolongation and quality of life, but to integrate them through a patient-defined understanding of meaningful time.

### 10.7. Interactions Between the Five Dimensions

The five dimensions of the model are interdependent. Objective time burden can increase subjective burden, but this relationship is moderated by psychological state, caregiver support, and existential meaning. For example, a patient may tolerate frequent visits if they provide symptom relief, hope, reassurance, and meaningful disease control. Another patient may experience fewer visits as intolerable if each visit triggers severe anxiety, disrupts childcare, or symbolizes loss of autonomy.

Psychological burden can also amplify objective burden. These interactions are mediated by complex neurobiological and behavioral pathways, highlighting the integration between mental and physical health systems. Anxiety may make waiting feel longer, depression may make recovery more difficult, and demoralization may make treatment time feel meaningless. Relational burden may intensify subjective burden if the patient feels guilty about caregiver time or family disruption. On the other side, strong support may reduce the perceived cost of care. Existential meaning may transform time toxicity: treatment time may feel acceptable when connected to a valued goal, but excessive when disconnected from the patient’s priorities.

Accordingly, the aim is not simply to reduce healthcare contacts but to maximize the value patients derive from the time invested in care.

### 10.8. Clinical Application of the Model

The model can be applied in routine advanced breast cancer care through a structured clinical assessment. At each major decision point, clinicians may evaluate the five dimensions and this assessment can be incorporated into shared decision-making, treatment planning, psycho-oncological referral, palliative care integration, and care pathway redesign. It can also guide research by suggesting which outcomes should be measured together. Advanced breast cancer is an ideal context for applying this model because its clinical course often involves prolonged treatment, multiple therapeutic transitions, recurrent imaging, evolving prognosis, and repeated negotiation between disease control and quality of life.

The model also helps avoid two opposing errors. The first is a purely oncological reductionism, in which treatment value is defined only by efficacy metrics. The second is an oversimplified convenience model, in which reducing visits is assumed to be inherently better. A psychiatry- and psychology-informed model recognizes that time in care can be burdensome, reassuring, necessary, meaningful, frightening, or oppressive depending on context. The clinical task is to understand which of these meanings applies to the individual patient.

In advanced breast cancer, the most patient-centered question may not be “How can we reduce time toxicity as much as possible?”, but rather “How can we ensure that the time required by care is proportionate, meaningful, psychologically tolerable, and aligned with the patient’s values?”. This reframing transforms time toxicity from a descriptive metric into a clinical and ethical tool.

The proposed model conceptualizes time toxicity as a multidimensional construct including objective, subjective, psychological, relational, and existential domains. This model is intended to support clinicians, researchers, and healthcare systems in evaluating advanced breast cancer care beyond traditional efficacy endpoints. It integrates psycho-oncology, palliative care, and health services research into a patient-centered framework. It therefore may provide a conceptual bridge between health services research, psycho-oncology, palliative care, and shared decision-making.

In doing so, it supports a more comprehensive definition of value in advanced breast cancer: one that includes not only how much time treatment can add, but how much meaningful time it can preserve. Prospective studies are needed to validate this framework before routine clinical implementation.

Table 4 summarizes the proposed domains of time toxicity, their potential indicators, and their implications for patient-centered care.

## 11. From Time Toxicity to Time-Concordant Care

### 11.1. Establishing Time Toxicity as a Standard Outcome

Future research should aim to establish time toxicity as a standard patient-centered outcome in advanced breast cancer. Although the concept has gained increasing attention in oncology, it remains inconsistently defined, measured, and reported. Current studies use heterogeneous indicators, including contact days, home days, days alive and out of hospital, healthcare utilization, hospital-free survival, treatment visits, and patient-reported treatment burden. These measures are valuable, but they are not interchangeable. A standardized taxonomy is needed to clarify which dimensions of time toxicity are being measured and for what purpose.

In advanced breast cancer, this standardization is particularly important because patients may receive treatment for prolonged periods across multiple lines of therapy. Future trials should report not only traditional endpoints such as overall survival, progression-free survival, response rate, and adverse events, but also the amount of time patients spend in healthcare-related activities. This should include scheduled and unscheduled visits, infusion time, imaging, laboratory monitoring, emergency department use, hospitalizations, travel time, waiting time, and recovery time. When feasible, caregiver time should also be included.

The integration of time toxicity into trial reporting would allow clinicians and patients to compare regimens not only according to efficacy and toxicity, but also according to the temporal burden required to obtain clinical benefit. This is especially important when treatments have similar efficacy but differ in route of administration, monitoring intensity, toxicity profile, or need for hospital-based care. A future advanced breast cancer trial could, for example, report progression-free survival together with the proportion of days spent in healthcare contact, patient-reported time burden, and days preserved at home.

Time toxicity should be viewed as complementary to, not competitive with, traditional oncological outcomes. Its value lies in improving the interpretation of benefit. A treatment that prolongs survival but requires frequent hospital attendance, prolonged recovery, and high emotional burden may have a different patient-centered value than a treatment with similar efficacy but lower temporal burden. Conversely, a time-intensive treatment may still be highly valuable if it produces substantial symptom relief, disease control, or meaningful survival prolongation.

### 11.2. Developing and Validating Specific Instruments

A major priority is the development and validation of specific instruments for measuring time toxicity. Current patient-reported outcome tools capture important domains such as physical symptoms, emotional functioning, social functioning, fatigue, pain, and global quality of life, but they rarely assess time burden directly. The EORTC QLQ-C30 and breast cancer modules provide essential information about health-related quality of life, but future instruments should more explicitly capture healthcare-related time, perceived loss of time, time spent waiting, travel burden, recovery time, disruption of daily activities, and subjective valuation of time.

The updated EORTC breast cancer module, QLQ-BR45, includes domains such as body image, future perspective, sexual functioning, sexual enjoyment, and breast satisfaction, making it more aligned with contemporary breast cancer survivorship and treatment experiences [84]. However, even disease-specific quality-of-life instruments do not fully capture time toxicity as an independent construct. Recent work on patient-reported outcome deterioration endpoints in cancer trials also highlights the importance of using patient-reported outcomes PROs not only as descriptive secondary outcomes, but as clinically interpretable measures of how treatment affects patient experience over time [85].

Future time toxicity instruments should include both objective and subjective components. Such instruments should be tested for reliability, validity, responsiveness to change, and interpretability.

In advanced breast cancer, instrument validation should include clinically and biologically diverse disease subgroups, including hormone receptor-positive, HER2-positive, and triple-negative disease. Studies should also represent patients receiving oral therapies, antibody–drug conjugates, or immunotherapy. Older adults, patients living far from cancer centers, individuals with psychiatric vulnerability, and those with limited social support should be adequately represented. Without this diversity, instruments may fail to capture the populations most affected by time toxicity.

### 11.3. Integrating Time Toxicity into Value Frameworks

Future work should also integrate time toxicity into oncology value frameworks. Current frameworks increasingly consider clinical benefit, toxicity, quality of life, and cost, but time burden is not always explicitly represented. The ESMO Magnitude of Clinical Benefit Scale is a validated and reproducible tool developed to assess the magnitude of benefit of anticancer treatments, and the 2025 version 2.0 update reflects ongoing efforts to refine how clinical benefit is evaluated in oncology [86]. Future value assessments should more systematically incorporate treatment-related time, especially in advanced disease.

Time toxicity could be incorporated into value frameworks in several ways. It could be reported as an independent patient-centered outcome. It could also be used to contextualize survival gains, for example by estimating the proportion of additional survival spent in healthcare settings. It could be integrated with quality-of-life and toxicity endpoints to generate a broader measure of patient-centered net benefit. Finally, it could inform shared decision-making tools by making visible the practical demands associated with each treatment option.

### 11.4. Digital Health and Remote Monitoring

Digital health technologies may play an important role in future time toxicity research and management.

Electronic patient-reported outcome systems, remote symptom monitoring, wearable devices, telemedicine platforms, and app-based supportive care tools can help capture real-time information on symptoms, functioning, adverse events, and treatment burden. In breast cancer, app-based digital capture of quality of life, adverse events, and treatment satisfaction has been shown to be feasible and acceptable, suggesting that digital tools may support longitudinal monitoring during treatment [87,88,89].

Recent randomized evidence in metastatic breast cancer is particularly promising. A multicenter randomized clinical trial in patients with ERBB2-positive metastatic breast cancer receiving trastuzumab deruxtecan found that electronic patient-reported outcomes with symptom tracking and vital-sign monitoring were associated with better quality-of-life scores at week 24 compared with usual care [88]. This supports the broader idea that digital monitoring may not only collect data, but also improve clinical management and patient experience when integrated into care pathways.

Digital health should be developed with attention to digital time toxicity. Remote monitoring may reduce travel and waiting time, but it can also create new obligations: completing questionnaires, managing devices, responding to alerts, coordinating local tests, and maintaining constant symptom vigilance. Future studies should therefore evaluate whether digital tools reduce or merely redistribute time burden. They should also assess usability, digital literacy, data fatigue, caregiver involvement, and psychological effects such as reassurance or increased hypervigilance.

### 11.5. Equity, Access, and Social Determinants of Time Toxicity

Future research should examine time toxicity as an equity issue. The same treatment schedule may impose very different burdens depending on transportation, income, employment flexibility, childcare, rural residence, disability, health literacy, immigration status, caregiver availability, and access to psycho-oncology. Patients with fewer resources may spend more time travelling, waiting, coordinating care, negotiating work absence, or managing fragmented services. They may also have fewer opportunities to choose time-saving alternatives.

Advanced breast cancer studies should therefore report contextual variables that influence time burden. These may include distance from treatment center, travel mode, employment status, caregiver availability, socioeconomic status, rural or urban residence, digital access, and availability of local services. Such data would allow researchers to identify groups at higher risk of disproportionate time toxicity and to design targeted interventions.

Equity-oriented solutions may include decentralized laboratory testing, local imaging partnerships, transportation support, flexible scheduling, patient navigation, telemedicine when appropriate, culturally sensitive communication, and integration of social work and psycho-oncology. Importantly, digital solutions should not widen disparities. Patients without reliable internet access, digital literacy, or caregiver assistance may be excluded from benefits if remote care becomes the default model without adequate support [82].

Psychiatric equity is also essential. Patients with depression, anxiety, cognitive impairment, trauma history, severe mental illness, or low social support may experience greater subjective time toxicity and greater difficulty navigating care. Future models should include psychological screening and support as part of time toxicity reduction, rather than treating mental health as separate from oncology pathway design.

### 11.6. Training Clinicians to Discuss Time

Another important future direction is clinician training. Many oncologists routinely discuss survival, response, adverse events, and treatment logistics, but time toxicity may remain implicit. Clinicians may not ask how patients experience hospital time, whether appointments interfere with meaningful activities, whether travel is sustainable, or whether the expected benefit feels proportionate to the temporal burden. As a result, patients may assume that time burden is not a legitimate topic for discussion.

Training programs should help clinicians incorporate time into shared decision-making. This includes learning how to explain treatment schedules clearly, estimate visit frequency and recovery time, discuss uncertainty, elicit patient values, and recognize psychological distress related to time burden. Communication should also address the emotional meaning of time, especially in advanced disease. Questions such as “What would you most like to preserve in your daily life?”, “How does this treatment schedule fit with your priorities?”, and “Does the time required by this treatment feel worthwhile to you?” can open clinically meaningful conversations.

Psychiatrists, psychologists, psycho-oncologists, oncology nurses, and palliative care clinicians can contribute to this training. Their expertise is particularly relevant when time toxicity intersects with depression, anxiety, demoralization, fear of progression, family conflict, or decisional fatigue. Future multidisciplinary education should therefore frame time toxicity not only as a logistical metric, but as a psychological and ethical issue.

### 11.7. Research Priorities for Advanced Breast Cancer

Several research priorities emerge for advanced breast cancer. Prospective studies should quantify time toxicity across different treatment classes, including endocrine therapy with CDK4/6 inhibitors, anti-HER2 therapy, antibody–drug conjugates, immunotherapy combinations, chemotherapy, and palliative radiotherapy. Studies should compare time toxicity across routes of administration, including intravenous, subcutaneous, oral, and hybrid care models. Research should evaluate whether interventions such as visit clustering, telemedicine, digital symptom monitoring, local testing, early palliative care, and psycho-oncological support reduce objective or subjective time burden.

Ongoing studies should investigate the relationship between time toxicity and psychiatric outcomes. Relevant outcomes include depression, anxiety, demoralization, scanxiety, fear of progression, insomnia, decisional fatigue, perceived autonomy, caregiver burden, and existential distress. This is especially important because the psychological impact of time toxicity may be clinically meaningful even when objective healthcare contact is modest. Trials should examine how patients trade survival benefit against time burden. Discrete choice experiments, qualitative interviews, mixed-methods studies, and embedded trial preference studies could clarify how patients value time across disease phases. These methods may help identify which patients prioritize maximal disease control, which prioritize time at home, and how preferences change with progression, symptoms, or cumulative treatment exposure.

Implementation research is needed. It is not enough to define and measure time toxicity; care systems must learn how to reduce it. Future interventions should be tested in real-world oncology settings, including academic centers, community hospitals, rural care networks, and digitally supported hybrid models. Outcomes should include feasibility, acceptability, safety, equity, patient satisfaction, quality of life, psychological distress, caregiver burden, and healthcare utilization.

### 11.8. Toward a Research Agenda Integrating Psychiatry and Oncology

The future study of time toxicity should be explicitly interdisciplinary. Oncology can quantify disease control, treatment efficacy, toxicity, and healthcare utilization. Psychiatry and psychology can clarify how time burden affects distress, identity, autonomy, coping, decisional capacity, and existential meaning. Palliative care can help align treatment with goals and preserve quality of life. Health services research can evaluate system-level inefficiencies and design time-saving interventions. Patient and caregiver involvement can ensure that research outcomes reflect lived experience rather than administrative convenience.

For advanced breast cancer, such an interdisciplinary agenda is particularly timely. The expanding therapeutic landscape has made treatment more effective but also more complex. Patients may live longer with metastatic disease, but they may also spend more time navigating care. The challenge for future research is to ensure that therapeutic progress does not inadvertently produce a life increasingly organized around medical demands.

## 12. Conclusions

Time toxicity is an emerging patient-centered dimension of advanced cancer care that includes both measurable healthcare-related time and its subjective impact on patients’ daily lives. In advanced breast cancer, this issue is particularly relevant because prolonged treatment exposure and repeated clinical reassessment may impose a substantial temporal burden alongside their therapeutic benefits [1,2,3,4,6].

Available evidence indicates that healthcare contact days, treatment visits, hospitalizations, travel, and recovery time do not fully capture patients’ experience of treatment-related time [15,59]. Psychological, relational, and existential factors may modify the perceived burden of care, although current evidence remains mainly observational and requires further validation.

The strength of evidence varies across the dimensions considered. Objective treatment schedules and healthcare utilization are supported by breast cancer-specific data in selected therapeutic contexts, whereas several psychological, relational, and existential dimensions are informed partly by broader oncology and psycho-oncology literature. These extrapolations are clinically plausible but require direct evaluation in advanced breast cancer populations.

Time toxicity should be considered a complementary dimension of treatment burden, alongside efficacy, safety, and quality of life. Patient-centered discussions should include not only expected benefits and adverse effects, but also the temporal demands and meaning of care.

The proposed model represents a hypothesis-generating framework intended to organize objective, psychological, relational, and existential dimensions of time burden and guide future research. Prospective studies are needed to validate standardized measures and evaluate whether interventions targeting avoidable time burden improve patient-reported outcomes.

Current evidence supports greater attention to time toxicity in advanced breast cancer but does not yet support routine clinical implementation of the proposed framework. Its value lies in promoting a broader understanding of treatment burden, including not only survival duration but also the quality and usability of patients’ time.

## Figures and Tables

**Table 1:** Components of time toxicity in advanced breast cancer care.

Domain of Time Toxicity	Examples in Advanced Breast Cancer	Possible Metrics	Psychological and Clinical Implications
Direct treatment time	Infusion visits, day-hospital attendance, subcutaneous injections, oral therapy monitoring, pre-treatment blood tests, post-infusion observation	Number of treatment visits; infusion duration; treatment-room time; contact days; days alive and out of hospital	May reduce autonomy and daily functioning; may influence preference for oral, subcutaneous, or less intensive regimens
Diagnostic and monitoring time	Imaging, laboratory tests, cardiac monitoring for anti-HER2 therapy, toxicity surveillance, biopsies, reassessment after progression	Number of scans; number of laboratory visits; monitoring frequency; waiting time for results	May trigger scanxiety, fear of progression, anticipatory anxiety, and difficulty planning future activities
Unplanned healthcare time	Emergency department visits, hospital admissions, urgent consultations, toxicity-related assessments, infections, febrile neutropenia, uncontrolled symptoms	Emergency visits; hospitalizations; unplanned contact days; toxicity-related visits	May increase loss of control, distress, perceived treatment burden, caregiver strain, and fear of clinical deterioration
Travel and logistical time	Transport to oncology center, parking, waiting rooms, administrative procedures, pharmacy access, coordination of appointments	Travel distance; travel time; number of separate trips; waiting time; appointment fragmentation	May disproportionately affect patients with rural residence, low income, poor mobility, limited support, or high caregiver dependence
Recovery time	Fatigue after treatment, nausea, pain, diarrhea, sleep disruption, cognitive exhaustion, recovery after hospitalization	Patient-reported recovery days; days unable to perform usual activities; symptom duration	May reduce meaningful time at home, social participation, work ability, and emotional resilience
Caregiver time	Accompanying patient, transport, medication management, symptom monitoring, communication with clinicians, family reorganization	Caregiver hours; work disruption; number of accompanied visits; caregiver-reported burden	May create family strain, patient guilt, relational stress, and hidden socioeconomic burden
Psychological time	Anticipatory anxiety, rumination, waiting for results, fear of progression, mental preparation for treatment, post-visit emotional recovery	Distress scales; fear of progression measures; patient-reported time burden; qualitative assessment	Captures the subjective occupation of time by illness and treatment; may be amplified by depression, anxiety, demoralization, or trauma history
Existential time	Perceived loss of meaningful time, inability to plan, disruption of life projects, awareness of limited future, conflict between survival and quality of time	Values-based interviews; meaning-centered assessment; patient priorities; goal-concordance discussions	Central to advanced disease; may influence treatment acceptance, refusal, or preference for less burdensome care

Note. This table is based on the conceptual distinction between objective healthcare utilization and subjective, psychological, relational, and existential dimensions of time burden.

**Table 2:** Psychiatric and psychological dimensions associated with time toxicity in advanced breast cancer.

Psychiatric or Psychological Dimension	Clinical Manifestations	How It May Amplify Time Toxicity	Implications for Decision-Making	Possible Interventions
Depression	Low mood, anhedonia, fatigue, hopelessness, psychomotor slowing, impaired concentration, sleep disturbance	Hospital visits and treatment schedules may be experienced as exhausting, meaningless, or disproportionate; recovery time may feel longer	May reduce active participation, impair comprehension, increase passivity, or lead to treatment refusal driven by hopelessness	Depression screening; psychiatric assessment; antidepressant treatment when indicated; psychotherapy; sleep and fatigue management
Anxiety	Excessive worry, somatic hypervigilance, panic symptoms, insomnia, reassurance-seeking	Waiting, scans, treatment changes, and monitoring intervals may become emotionally intolerable; medical time expands through anticipation	May increase demand for frequent monitoring or impair tolerance of uncertainty	Psychoeducation; cognitive-behavioral strategies; anxiolytic treatment when appropriate; structured communication; predictable follow-up
Fear of progression/scanxiety	Fear of disease worsening, distress before imaging, anxiety while waiting for results, catastrophic thinking	Short medical procedures may dominate psychological life for days or weeks; scan intervals structure emotional time	May influence preference for intensive surveillance or avoidance of information	Timely communication of results; scanxiety-focused interventions; coping skills; psychological support before and after imaging
Demoralization	Helplessness, loss of meaning, subjective incompetence, disheartenment, existential despair	Treatment-related time may be perceived as evidence of loss of dignity, dependency, or futility	May lead to ambivalence, desire to stop treatment, or requests for less burdensome care	Meaning-centered psychotherapy; dignity therapy; supportive-expressive therapy; early palliative care; psychiatric evaluation
Decisional fatigue	Cognitive exhaustion, indecision, avoidance, difficulty comparing options, dependence on clinician or family	Repeated treatment choices and care coordination increase mental burden beyond actual hospital time	May impair shared decision-making and increase vulnerability to passive or inconsistent choices	Simplified communication; decision aids; staged discussions; values clarification; caregiver involvement when desired
Trauma-related vulnerability	Hyperarousal, avoidance, intrusive memories, distress during procedures, loss-of-control reactions	Hospital environments, invasive procedures, scans, or bodily monitoring may reactivate prior traumatic experiences	May lead to avoidance, non-adherence, intense distress, or disproportionate fear of procedures	Trauma-informed care; predictable procedures; consent-sensitive communication; psychological therapy; psychiatric treatment if needed
Insomnia and fatigue	Non-restorative sleep, daytime exhaustion, reduced concentration, irritability	Treatment days, waiting, travel, and recovery become more burdensome; resilience to healthcare demands decreases	May reduce tolerance of treatment burden and impair understanding of complex information	Sleep assessment; behavioral sleep interventions; medication review; fatigue management; symptom control
Low perceived control	Helplessness, frustration, anger, dependence, inability to plan	Time feels allocated by the healthcare system rather than chosen by the patient	May increase distress and reduce sense of agency in treatment decisions	Shared decision-making; flexible scheduling; explicit discussion of patient priorities; restoration of choice where possible
Social isolation/poor support	Limited transport, lack of emotional containment, difficulty managing appointments, loneliness	Objective time burden becomes harder to manage and subjectively more threatening	May constrain treatment options and increase inequity	Social work referral; caregiver mapping; patient navigation; community support; telemedicine when appropriate
Caregiver strain	Family exhaustion, work disruption, relational tension, patient guilt	Time toxicity is transferred to family members and may become a relational burden	May influence treatment preferences, sometimes indirectly or silently	Caregiver assessment; flexible scheduling; caregiver psychoeducation; family meetings; respite and social support

Note. These dimensions may act as moderators between objective healthcare-related time and subjective time burden. Their identification can help clinicians distinguish logistical burden from treatable psychiatric or psychological distress.

**Table 3:** Strategies to reduce time toxicity in advanced breast cancer.

Strategy	Clinical Rationale	Expected Impact on Time Toxicity	Psychological Benefit	Implementation Challenges
Use of oral therapies when clinically appropriate	Reduces need for infusion visits and day-hospital attendance	Decreases direct treatment time and travel; shifts some care to home	May increase autonomy and daily-life continuity	Requires adherence support, toxicity education, laboratory monitoring, and attention to anxiety or cognitive burden
Subcutaneous formulations	Shorter administration compared with intravenous treatment; may reduce chair time	Reduces treatment-room time and potentially overall clinic duration	May improve convenience, comfort, and perceived control	Benefit depends on redesign of the entire visit; monitoring and travel may still remain
Shorter infusion protocols when safe	Minimizes time spent in treatment units	Reduces direct treatment time and waiting-related burden	May reduce fatigue, frustration, and disruption of daily routines	Requires protocol validation, staffing coordination, and safety monitoring
Appointment clustering/one-stop clinics	Combines blood tests, consultations, imaging, pharmacy, and treatment on the same day	Reduces number of separate trips and fragmented healthcare contacts	Improves predictability and planning; reduces logistical stress	Requires institutional coordination and scheduling flexibility
Reduction in avoidable waiting time	Waiting is a major source of subjective and psychological time toxicity	Reduces non-value-added time in care settings	May reduce anxiety, anger, helplessness, and perceived disrespect	Requires workflow redesign, communication systems, and real-time delay management
Telemedicine for selected visits	Replaces visits not requiring physical examination, infusion, or urgent assessment	Reduces travel and waiting-room exposure	May preserve energy and time at home; may reduce hospital-related distress	May increase digital workload; not appropriate for all patients; requires digital access and literacy
Local laboratory testing and decentralized monitoring	Allows selected assessments closer to home	Reduces travel burden and dependence on tertiary centers	May increase autonomy and reduce caregiver burden	Requires data integration, communication with local services, and safety protocols
Digital symptom monitoring	Enables earlier detection of adverse events and remote triage	May prevent avoidable emergency visits or hospitalizations	Provides reassurance and structured communication	Risk of digital time toxicity, alert fatigue, and increased self-monitoring anxiety
Early palliative care integration	Improves symptom control, communication, goal clarification, and anticipatory planning	May reduce crisis-driven healthcare use and non-beneficial treatment time	Supports meaning, dignity, coping, and family communication	Persistent stigma; variable access; need for early referral pathways
Psycho-oncological screening and intervention	Identifies depression, anxiety, scanxiety, demoralization, insomnia, and decisional fatigue	Reduces subjective time burden even when objective visits cannot be avoided	Improves emotional regulation, decisional capacity, and perceived control	Limited availability of psycho-oncology services; need for routine screening
Structured scan-result communication	Reduces prolonged uncertainty after imaging	Decreases emotional occupation of time around scans	May reduce scanxiety and fear of progression	Requires timely reporting, clinician availability, and clear communication pathways
Caregiver assessment and support	Recognizes caregiver time as part of total treatment burden	Reduces hidden family time toxicity and improves sustainability of care	May reduce patient guilt and relational tension	Caregiver burden is often invisible; requires family-aware care models
Patient navigation	Supports appointment coordination, education, and access to resources	Reduces administrative burden and fragmented care	May reduce confusion, helplessness, and anxiety	Requires trained staff and integration into oncology pathways
Equity-oriented scheduling and transport support	Addresses disproportionate burden in patients with low resources, rural residence, or poor support	Reduces travel-related and socioeconomic time toxicity	May improve access, adherence, and perceived fairness	Requires institutional resources and identification of high-risk patients
Values-based shared decision-making	Aligns treatment burden with patient priorities and goals	Ensures that time spent in care is proportionate and meaningful	Supports autonomy, dignity, and goal-concordant care	Requires communication training and sufficient consultation time

Note. These strategies should be individualized according to disease subtype, treatment line, prognosis, psychiatric vulnerability, caregiver resources, and patient preferences.

**Table 4:** Clinical summary of the main domains associated with time toxicity in advanced breast cancer.

Domain	Definition and Principal Components	Potential Indicators or Assessment Areas	Proposed Clinical Implications
Objective time toxicity	Measurable time devoted to accessing, receiving, monitoring, and recovering from cancer care. It includes treatment visits, investigations, travel, waiting, hospitalizations, unplanned contacts, and recovery days.	Healthcare contact days; visit frequency and duration; travel and waiting time; hospital admissions; emergency visits; days required for treatment-related recovery.	May be considered alongside efficacy, safety, and health-related quality of life when comparing treatment options. Potentially avoidable temporal burden may be reduced through care coordination, appointment clustering, decentralized services, and less time-intensive treatment modalities when clinically appropriate.
Subjective time burden	The patient’s perception of how cancer care occupies, disrupts, or constrains daily and future time. Equal objective demands may be experienced differently across patients.	Perceived interference with daily activities; difficulty planning; loss of spontaneity or autonomy; perceived proportionality between treatment burden and expected benefit; perceived value of time spent in care.	Direct discussion of time preferences may help identify whether a proposed treatment schedule is compatible with the patient’s priorities, routines, and desired use of time.
Psychological and psychiatric modifiers	Emotional or psychiatric factors that may influence how healthcare-related time is perceived and tolerated, including depression, anxiety, fear of progression, demoralization, trauma-related symptoms, insomnia, and decisional fatigue.	Distress screening; depressive and anxiety symptoms; fear of progression; demoralization; sleep disturbance; cognitive overload; prior psychiatric history; coping resources.	Symptoms may affect quality of life, symptom perception, communication, treatment engagement, adherence, decision-making, and healthcare utilization. Their presence does not establish a direct causal effect on tumor progression or survival. Appropriate psycho-oncological or psychiatric assessment may support treatment participation and emotional adaptation.
Caregiver and relational burden	Time and practical responsibilities assumed by family members or informal caregivers, together with the effects of treatment schedules on family relationships and social roles.	Accompanied visits; transport and coordination responsibilities; caregiver work disruption; medication and symptom management; family reorganization; caregiver-reported burden.	Care planning may consider caregiver availability and burden without displacing the patient’s autonomy. Telemedicine, oral treatment, or decentralized care may reduce some demands while shifting others to the home or caregiver.
Patient-centered clinical implications	Integration of objective burden, subjective experience, psychological vulnerability, caregiver circumstances, and patient preferences into treatment discussions.	Patient priorities; acceptable healthcare-related time; activities the patient wishes to preserve; treatment goals; expected benefit; route and frequency of treatment; clinical and logistical feasibility.	These domains may provide a preliminary structure for shared decision-making and future research. They should not currently be used as a validated score, risk-stratification system, or prescriptive clinical algorithm. Prospective advanced breast cancer-specific validation is required.

Note. This table provides a concise overview of domains and possible clinical implications discussed in the text.

## Data Availability

No new data were created or analyzed in this study. Data sharing is not applicable.
